# Heterogeneity in lung cancers by single‐cell DNA sequencing

**DOI:** 10.1002/ctm2.1388

**Published:** 2023-08-30

**Authors:** Li Zhang, Lingxi Chen, Shuai Cheng Li, Mengyao Wang, Chaohui Li, Tingting Song, Yinyun Ni, Ying Yang, Zhiqiang Liu, Menglin Yao, Bairong Shen, Weimin Li

**Affiliations:** ^1^ Department of Pulmonary and Critical Care Medicine Institute of Respiratory Health State Key Laboratory of Respiratory Health and Multimorbidity Frontiers Science Center for Disease‐related Molecular Network Precision Medicine Key Laboratory of Sichuan Province West China Hospital West China School of Medicine Sichuan University Chengdu China; ^2^ Department of Computer Science City University of Hong Kong Kowloon China; ^3^ Institutes for Systems Genetics Frontiers Science Center for Disease‐Related Molecular Network West China Hospital Sichuan University Chengdu China

Dear Editor,

Lung carcinoma genomes are heterogeneous. We probe heterogeneity origins by sequencing 13,343 single‐cell genomes from seven lung adenocarcinomas (LUAD), seven lung squamous cell carcinomas (LUSC), and two small‐cell lung carcinomas (SCLC). Our findings reflect lung tumors holding huge subclone diversity on copy number variations (CNVs) and complex structure variations (cSVs).

Lung cancer tops global cancer deaths, with intra‐tumor heterogeneity (ITH) contributing to recurrence and resistance.^1^ Single‐cell DNA sequencing (scDNA‐Seq)^2^ provides a precise ITH perspective by profiling individual cells in multiple cancers,^3^, ^4^, ^5^, ^6^ but limited in lung cancer. Most lung cancer studies examine subclonal CNVs,^7^ leaving cSV’ ITH, and their role in lung cancer progression remains incompletely understood.^8^, ^9^

This study analyzed 13,343 single‐cell genomes from 16 lung tumors: 7 LUAD, 7 LUSC, and 2 SCLC (Figure 1, Table S1, Supporting Information Tables and Supporting Information Methods). We investigated the CNV landscape across all tumors. Tumor cell groups were obtained from hierarchical clustering (HC) using cell ranger‐DNA.^6^ We assigned cells grouped by a leaf node in the HC cut‐dendrogram as cell clusters; that is, cells inside one cell cluster sharing similar CNVs. We identified 16 to 33 cell clusters per tumor (Figures S1–S3), yielding three to nine subclones per tumor and 72 subclones overall (Table S2). All tumors display polyclones, meaning they have at least two subclones. Subclones are denoted by dominant amplified (A), diploid (D), or lost (L) copy numbers (Table S2). “LX” indicates subclone has loss of heterozygosity in chromosome X. The largest subclone populates 1,273 diploid cells (LUAD03T‐D), whilst we detected 23 small cell populations, that is, subclones under 10 cells (Table S2). We calculated the Gini index per subclone, with higher values reflecting greater CN dispersion across genomic regions in the subclone. Overall, cell numbers and Gini indices vary between subclones, illustrating CNV tumor heterogeneity in LUAD, LUSC, and SCLC (Figure 1 and Table S2). Moreover, hierarchical clustering of subclone CNVs revealed inter‐patient similarities (Figures S4 and S5, Table S3 and Supporting Information Results).

**FIGURE 1:**
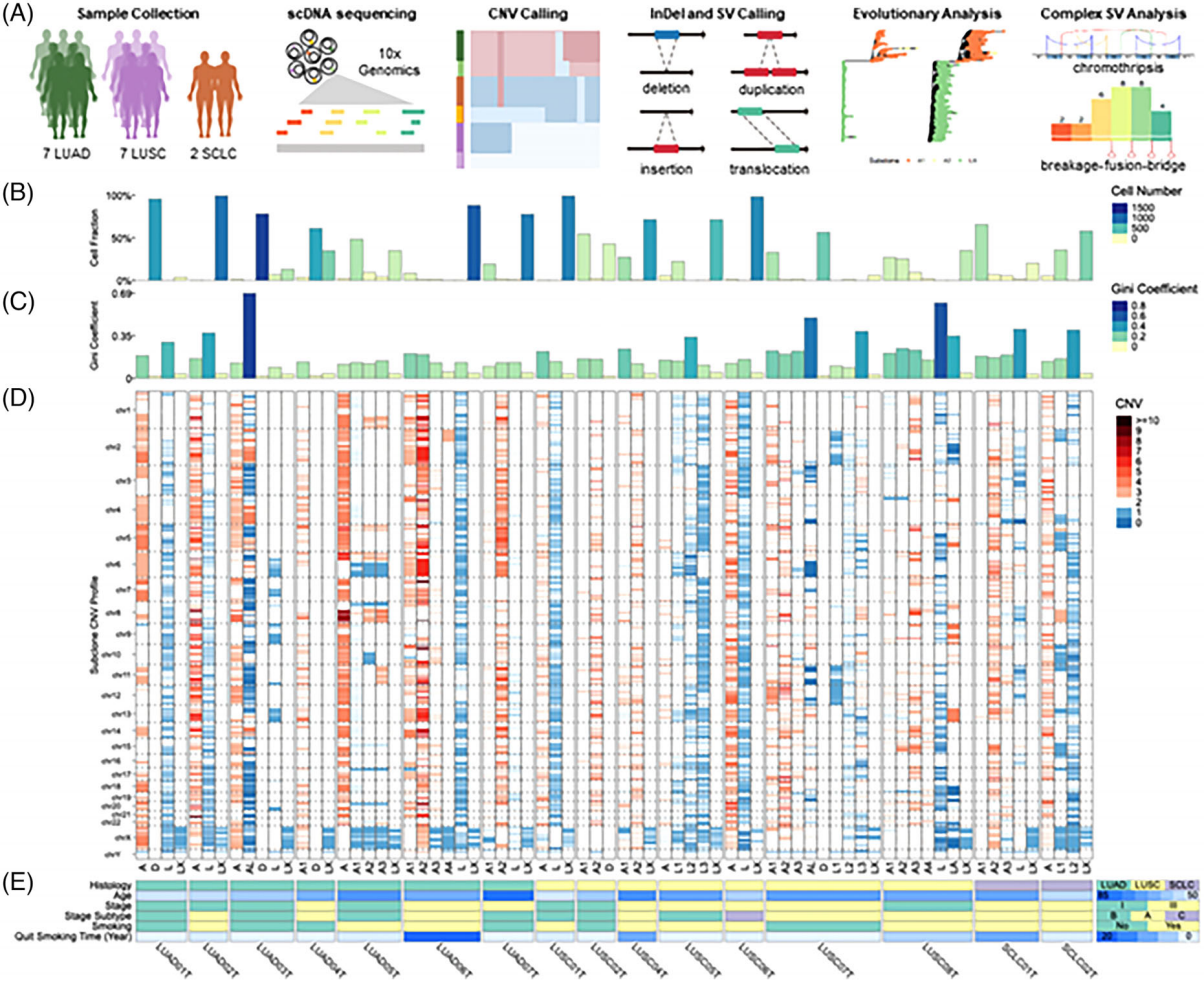
Spectrum of subclone and copy number variations (CNVs) on lung cancer tumors. (A) The schematic illustration of the study design. (B) Fraction and number of non‐noisy single cells identified in tumor subclones across lung tumors. (C) Gini indices of CNVs in tumor subclone across lung tumors. (D) CNVs of tumor subclone across lung tumors. (E) Clinical information across lung tumors.

Punctuated copy number evolution (PCNE) hypothesizes subclonal CNVs arise in short bursts of crisis while branching copy number evolution (BCNE) hypothesizes subclonal CNVs are intermediate accumulated over evolution.^3^, ^4^, ^5^ We manually infer CNV evolutionary trees (Figure 2A) from tumor cell‐ and subclone‐level phylogenies, alongside subclone‐level consensus CNVs (Figures S6–S8). We posit most lung tumors experience PCNE, with certain subclones subsequently undergoing BCNE (Figures S6–S8 and Supporting Information Methods). We hypothesize copy number evolution begins in normal tissue and then diverges into A‐, D‐ or L‐dominated subclones, which may continue evolving. We suffixed indices to subclone names to indicate their occurrence in the evolution process (e.g. A2 occurs after A1). All lung tumors exhibit PCNE signatures, except LUAD02T and LUSC01T, forming one dominant group with minor clones under ten cells. Likewise, six lung tumors (LUAD05T, LUAD06T, LUAD07T, LUSC07T, LUSC08T and SCLC01T) show BCNE evidence derives post‐PCNE subclones (≥10 cells) (Figures S6–S8). SCLC02T‐A features *MYC* and *ASCL1* amplification, while SCLC02T‐L exhibits *MYCN* amplification. Both *MYC* and *MYCN* promote SCLC in mice.^10^ Distinct amplified genes in subclones A and L suggest differing evolutionary paths. *APC* is specifically amplified in LUAD06T‐A2 and LUAD07T‐A2. *APC* amplification significantly correlates with better progression‐free survival of TCGA‐LUAD (Figure 2B). *CEP89* and *FAT3*, frequently amplified in LUAD and LUSC, carry prognostic significance in TCGA‐LUAD (Figure 2C,D).

**FIGURE 2:**
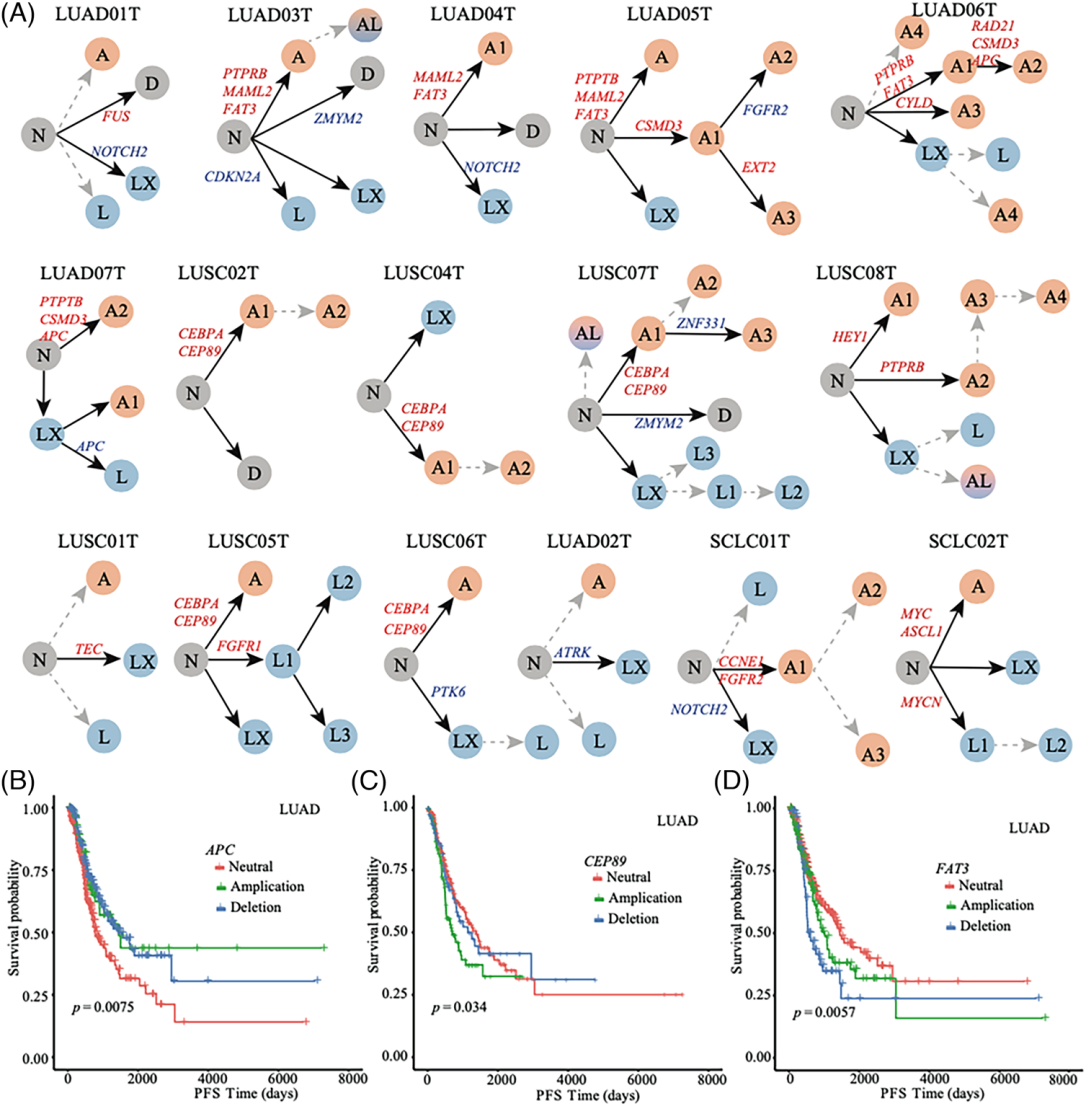
Copy number evolutionary interpretation on lung cancer tumors. (A) CNV subclone evolutionary tree for 16 lung cancer tumors. We marked key cancer‐related genes for each branch. CNV amplified and deleted genes are colored in blue and red, respectively. (B–D) Progression‐free survival curves of TCGA‐ LUAD patients stratified by CNV of *APC* (B), *CEP89* (C) and *FAT3* (D).

We subclone‐level annotated InDels, SVs and cSVs in lung tumors. Figure 3A displays highly mutated samples (LUAD03T, LUAD04T, LUSC04T and LUSC05T) alongside samples with fewer mutations (LUAD05T, LUAD06T and LUSC02T). Subclone‐level deletions dominate across cohorts (Table S4). Many genetic alterations (10∼70%) co‐exist in tumor subclones formed by PCNE (Figure 3B, Figures S9 and S10, Table S5, and Supporting Information Result). These findings indicate most genome aberrations occur before or during CNV bursts. We constructed single‐cell phylogenies using CNVs and SVs to validate the speculation. SV‐ plus CNV‐based phylogenies match CNV‐derived phylogenies, indicating pre‐PCNE genome variations (Figures S11–S13). Moreover, diverse intra‐tumor genetic alterations are evident across cohorts. Several tumor subclones harbor exclusive InDels, SVs, or cSVs, consistent with the post‐punctuated CNV burst BCNE hypothesis. Furthermore, we detected two breakage‐fusion bridges in LUSC, duplicating oncogenes *PLA2G4A* and *GBE1*, which may occur accompanying PCNE (Figures S14 and S15 and Supporting Information Results). Interestingly, InDels, SVs, and cSVs recurrently hit Human leukocyte antigens (HLA) genes in subclones (Figure 3C and Figure S16 and Table S6). We detected cSVs harboring in MHC‐II genes. LUSC shows more SV breakpoints in the *HLA‐DRB* gene than LUAD. These findings suggest cSVs in MHC‐II genes coincide with or precede CNV bursts (Figures S17–S20 and Supporting Information Results). Despite tumor heterogeneities, we identified recurrent genetic alterations in LUAD‐ or LUSC‐related genes (Figure 3C and Supporting Information Results).

**FIGURE 3:**
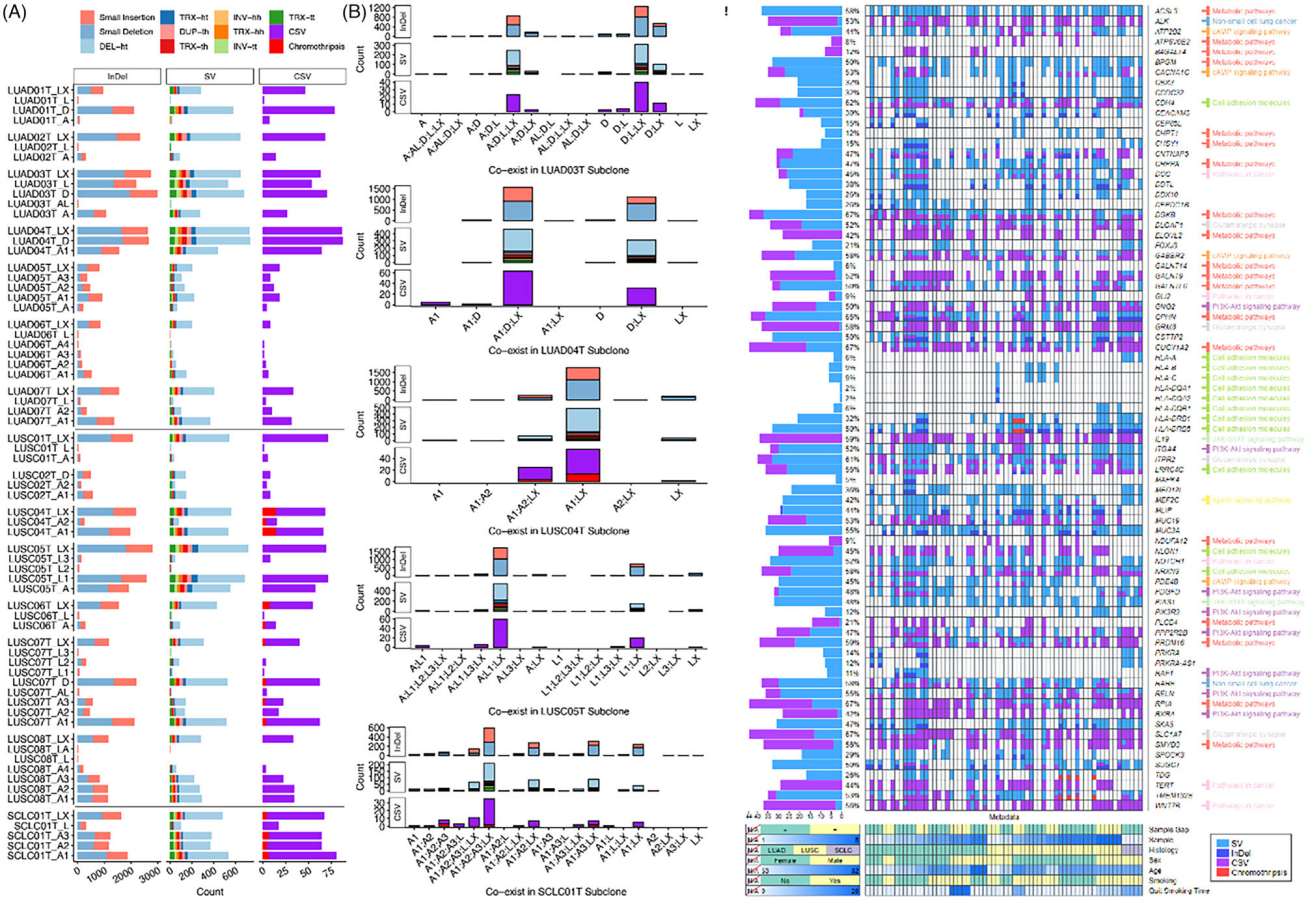
Subclone‐level genome aberration landscape across the cohort. (A) The number of InDel (small insertion and deletion), SV (DEL‐ht, TRX‐ht, DUP‐th, TRX‐th, INV‐hh, TRX‐hh, INV‐tt and TRX‐tt), and CSV (Chromothripsis and other CSV) in each tumor subclone. (B) The frequency of co‐existence of InDel, SV, and CSV in different combinations of subclones for LUAD03T, LUAD04T, LUSC04T, LUSC05T and SCLC01T. The top, middle, and bottom layer presents InDel, SV, and CSV, respectively. In LUAD03T, the most genetic alterations coexist in subclones formed by PCNE (28.58% for D, L, LX and A; 40.81% for D, L and LX). In LUAD04T, subclones D, A1, and LX (formed by PCNE) share 57.34% common genetic alterations, subclone group D and LX have 40.14% common genetic alterations. In LUSC04T, the PCNE‐produced subclones LX and A1 have 78.10% common genetic alterations, as does the minor subclone A2 (eight cells, 12.03% for A1, A2 and LX). In LUSC05T, 61.15% genetic alterations were recurrently observed in subclones A, LX, and L1 derived from PCNE. Subclone groups L1 and LX also share 23.68% common alterations. In SCLC01T, PCNE derived LX and A1, BCNE derived A2 and A3 also shares a plenty of genetic alterations (10.93% for A1 and LX; 15.60% for A1, A3, and LX; 14.18% for A1, A2 and LX; 31.92% for A1, A2, A3, and). The minor subclone L with six cells also shares several genetic alterations with the other four subclones. (C) Landscape of genetic alterations identified in multiple signal pathways associated with lung cancer throughout the cohort at the subclone level. The right panel demonstrates the subclone mutational frequency per gene for the cohort. The corresponding genes, gene families, and pathways are annotated on the right side. The bottom panel exhibits clinical metadata.

In brief, we used 10x scDNA‐Seq to reveal extensive subclone diversity in CNVs and cSVs across LUAD, LUSC and SCLC. The number of cell clusters and subclones aligns with previous breast cancer scDNA‐Seq studies.^3^, ^4^, ^5^ We suggest PCNE in three lung cancer subtypes, characterized by early genomics gains and losses, followed by BCNE. Two breakage‐fusion‐bridges duplicating oncogenes *PLA2G4A* and *GBE1* were detected in LUSC, potentially linked to PCNE. cSVs are identified in lung cancers, especially with high frequency (75%) in LUSC, affecting two MHC‐II genes (*HLA‐DRB5* and *HLA‐DRB1*). Evolutionary analysis suggests these cSVs may occur before or during PCNE. Hence, our findings reflect extensive subclone diversity in lung tumors concerning CNVs and cSVs.

One study limitation is subclone detection dependent on 10x scDNA‐Seq cell profiling,^6^ partially repenting subclone diversity in tumors. Average single‐cell coverage was low (Supporting Information Tables), potentially concealing SVs and cSVs in minor subclones. The issue is prevalent in scDNA‐Seq, we aim to sequence more lung tumors to enrich findings. PCNE and BCNE hypotheses rely solely on observing cell phylogenies and subclone CNVs. Looking forward, we plan to mathematically model PCNE and BCNE processes for quantitative answers.

## CONFLICT OF INTEREST STATEMENT

Not applicable.

## FUNDING INFORMATION

This work was supported by National Natural Science Foundation of China (Nos. 81974363, 81772478 to Li Zhang; 81871890, 91859203, 92159302 to Weimin Li), CAMS Innovation Fund for Medical Science (No: 2019TX310002 to Weimin Li), Science and Technology Project of Sichuan (2022ZDZX0018 to Weimin Li), 1.3.5 Project for Disciplines of Excellence, West China Hospital, Sichuan University (ZYGD22009 to Weimin Li), and Key basic research projects of Shenzhen Science and Technology Innovation Commission (JCYJ20200109143216036 to Shuai Cheng Li).

## Supporting information

Supporting Information

Supporting Information

## Data Availability

The data that support the findings of this study are available at https://doi.org/10.57760/sciencedb.08329.
