# Supplementary material for: Heterogeneity in lung cancers by single‐cell DNA sequencing
Source: Clin Transl Med. 2023 Aug 30;13(9):e1388. doi: 10.1002/ctm2.1388 (PMC10468563; doi:10.1002/ctm2.1388)
Supplement: Supplementary file 1 — Supporting Information [file CTM2-13-e1388-s002.docx]

**Heterogeneity in Lung Cancers by single-cell DNA sequencing**

-

Supplementary Materials

Table of Contents

[1 Supplementary Background 3](#_Toc142160251)

[2 Supplementary Results 5](#_Toc142160252)

[2.1 Copy number intra-heterogeneity of samples 5](#_Toc142160253)

[2.2 Punctuated copy number evolution of lung cancer tumors 5](#_Toc142160254)

[2.3 Most structural aberrations may occur before or after the punctuated copy number evolution. 6](#_Toc142160255)

[2.4 Complex rearrangements before or upon PCNE recurrently hit MHC II genes on LUSC tumors on subclone-level 7](#_Toc142160256)

[2.5 Similar and recurrent copy number and structure variation substructures across different patients for lung tumor samples 8](#_Toc142160257)

[3 Supplementary Discussion 10](#_Toc142160258)

[4 Supplementary Methods 13](#_Toc142160259)

[4.1 Sample collection 13](#_Toc142160260)

[4.2 10x CNV sequencing 13](#_Toc142160261)

[4.3 CNV analysis 13](#_Toc142160262)

[4.4 SV analysis 14](#_Toc142160263)

[4.5 Evolutionary analysis and interpretation 14](#_Toc142160264)

[4.6 CNV prognostic analysis 15](#_Toc142160265)

[5 Supplementary Figures 16](#_Toc142160266)

[Figure S1: Copy number substructure of seven LUAD tumors. 16](#_Toc142160267)

[Figure S2: Copy number substructure of seven LUSC tumors. 17](#_Toc142160268)

[Figure S3: Copy number substructure of two SCLC tumors. 18](#_Toc142160269)

[Figure S4: Subclone-level copy number landscape across the cohort. 19](#_Toc142160270)

[Figure S5: Subclone-level focal gains and losses across the cohort. 20](#_Toc142160271)

[Figure S6. Copy number evolutionary interpretation on lung cancer tumors. 21](#_Toc142160272)

[Figure S7: Copy number evolutionary interpretation of additional five LUAD lung tumors. 22](#_Toc142160273)

[Figure S8: Copy number evolutionary interpretation of additional five LUSC lung tumors. 23](#_Toc142160274)

[Figure S9: The frequency of genome aberrations co-existed in multiple subclones for additional five LUAD tumors. 24](#_Toc142160275)

[Figure S10: The frequency of genome aberrations co-existed in multiple subclones for additional five LUSC tumors. 25](#_Toc142160276)

[Figure S11: Copy number and structural variation evolutionary analysis of seven LUAD lung tumors. 26](#_Toc142160277)

[Figure S12: Copy number and structural variation evolutionary analysis of seven LUSC lung tumors. 27](#_Toc142160278)

[Figure S13: Copy number and structural variation evolutionary analysis of two SCLC lung tumors. 28](#_Toc142160279)

[Figure S14: Complex BFB event on chr1 for LUSC08T. 29](#_Toc142160280)

[Figure S15: Complex BFB event on chr3 for SCLC01T. 30](#_Toc142160281)

[Figure S16. Subclone-level heterogeneity of complex rearrangements on MHC II genes. 31](#_Toc142160282)

[Figure S17: The subclone-level of HLA-DRB genes associated complex rearrangements in LUAD tumors. 32](#_Toc142160283)

[Figure S18: The subclone-level of HLA-DRB genes associated with complex rearrangements in LUSC tumors. 33](#_Toc142160284)

[Figure S19: The subclone-level of HLA-DRB genes associated with complex rearrangements in SCLC tumors. 34](#_Toc142160285)

[Figure S20: The expression level of HLA-DRB1 and HLA-DRB5 in additional LUAD and LUSC case-control cohorts. 35](#_Toc142160286)

[6 References 36](#_Toc142160287)

# Supplementary Background

Lung cancer is the most common and lethal tumor worldwide^1^, and the most common pathological type of it is non-small-cell lung cancer (NSCLC), including lung adenocarcinoma (LUAD) and squamous cell cancer (LUSC)^2^. Despite multimodal clinical advances and efficacy, NSCLC remains an aggressive disease with a high risk of recurrence or resistance to therapies^3,4^. Small-cell lung cancer (SCLC), which presents around 15% of lung cancer instances, also has high recurrences^5^.

Studies have suggested that intra-tumor heterogeneity (ITH) leads to the recurrence and resistance of cancer. The most common approach to studying the ITH employs bulk DNA sequencing^6,7^. However, the bulk DNA-Seq intermixes tumor cells in individual lesions *en masse* during sequencing. The characteristics of rare subclone tumor cells are submerged in the dominant cell population, which conceals intra-tumor heterogeneity^8^. Single-cell DNA-sequencing (scDNA-Seq) resolves this hurdle by giving each cell a unique identifier during the DNA profiling^9–11^. scDNA-Seq has discriminated tumor subclones, built tumor clonal evolution history, and inferred the cause of cancer recurrence and therapeutic resistance in multiple cancers, including breast cancer^9,12–14^, acute myeloid leukemia^15,16^, colorectal cancer^17^, gastric cancer^11^, melanoma cancer^8^, etc. A series of single-cell genomic studies reported that breast cancers underwent punctuated copy number evolution (PCNE) processes other than gradual evolution modes^9,18–20^. However, single-cell genomic applications in lung cancers have been restricted to circulating tumor cells (CTC) in SCLC. The patterns of CTC copy number variations in LUAD can be different from those of SCLC^21^. Distinct copy-number profiles have been found in chemo-sensitive and refractory patients with SCLC^22^, and the evolution and progression history of CTC in SCLC has been inferred^23^. However, there lacks comprehensive study of intra-tumor heterogeneity and clonal evolution history in primary, solid NSCLC nodules at single-cell resolution.

Most genome heterogeneity studies in lung cancer focus on how single nucleotide variations (SNV) and copy number variations (CNV) changed in tumor subclones. Jamal-Hanjani et al. found that 75 of 100 NSCLC tumors shared heterogeneous driver mutations in PIK3GA and NF1, which occurred at the later stage during cancer evolution^6^. Somatic copy-number aberrations, gains on CDK4, FOXA1, and BCL11A, had a strictly positive association with ITH, and the amplified heterogeneity of CNV was related to recurrences in NSCLC ^6^. In alternative genome structural aberrations, 74% (102/138) of landmark fusion oncogenes, namely, EML4-ALK, CD74-ROS1, and KIF5B-RET, were generated with complex genome rearrangement, or complex structure variation (cSV), such as chromothripsis in a Korean LUAD cohort^24^. Breakage-fusion-breakage (BFB) events, another cSV with hallmarks of stair-like copy number gains, are identified in LUAD as well^25,26^. However, the intra-tumor heterogeneity of cSV, which is critical for lung cancer progression, has yet to be identified in lung cancers.

The intra-tumor heterogeneity and subclone evolution that occur on primary solid NSCLC nodules in single-cell resolution remain unexplained. The single-cell intra-tumor heterogeneity in structural variations and complex rearrangements, and their contribution to the tumor progress in LUAD, LUSC, and SCLC, remains unclear. We applied the 10x single-cell CNV solution^11^ to sixteen lung cancer samples, including seven LUAD, seven LUSC, and two SCLC, to address these issues. We harvested 13,343 non-noisy single cells and obtained 16 to 33 cell clusters (sub-subclones), where each tumor was annotated into 3 to 9 distinct subclones. The subclones within a sample have huge diversities in copy number variations and complex structural variations, whilst there exist subclones that have similar and recurrent copy number substructures across patients. Clonal analysis indicates that lung tumors undergo a punctuated copy number evolution (PCNE) event, followed by a branching evolution (BCNE) process. We detected two breakage-fusion-bridge events on lung squamous cell carcinomas that duplicate the oncogenes GBE1 and PLA2G4A separately. We found that in lung cancers, especially in squamous tumors with high frequency, i.e., 75% (6/8), complex rearrangements shattered two major histocompatibility complex (MHC) genes, HLA-DRB5 and HLA-DRB1. Further evolutionary analysis suggests that these complex rearrangements may occur before or at the time of the PCNE process.

# Supplementary Results

## Copy number intra-heterogeneity of samples

In this study, we obtained tumor samples from sixteen patients with primary lung tumor samples who had not undergone neoadjuvant therapy in West China Hospital, including seven lung adenocarcinoma (LUAD) tumor samples (LUAD01T-LUAD07T), seven lung squamous cell carcinoma (LUSC) tumor samples (LUSC01T, LUSC02T, and LUSC04T-LUSC08T), and two small cell lung cancer (SCLC) tumor samples (SCLC01T and SCLC02T; Figure 1 and Supplementary Table S1). We dissociated and profiled the cells using the 10x Genomics Single Cell CNV Solution^11^. Then we adopted the official 10x Genomics tool, cellranger-dna^11^, to profile the CNV for each cell (Supplementary Fig. S1-S3). The genome-wide copy number profiles were calculated at 20-kb resolution. In total, we have detected 13,343 single cells (7243, 5061, and 1039 cells for LUAD, LUSC, and SCLC, respectively).

First, we investigated the CNV landscape across all tumor samples. Cells that share similar CNV profiles form cell clusters and subclones per sample. We identified 16 to 33 cell clusters in each tumor (Supplementary Fig. S1-S3), where “cluster” is a leaf node consisting of a group of cells in the cut dendrogram of hierarchical clustering; that is, groups of cells sharing similar CNV profiles. Then, we manually assigned the cell clusters to subclones in each tumor according to their collapsed consensus CNV profile, which resulted in three to nine subclones in each tumor and to 72 tumor subclones in the sixteen tumors (Figure 1B and Supplementary Table S2). All tumors display polyclones; that is, each tumor sample consists of three to nine subclones, leading 72 tumor subclones from the sixteen tumor samples (Figure 1 and Supplementary Table S2). Following the convention, we denoted subclones dominated by amplified, neutral, and lost copy numbers with “A”, “D”, and “L”, respectively (Figure 1). “LX” refers to that subclone is loss of heterozygosity (LOH) in chromosome X. The largest subclone populates 1,273 diploid cells (LUAD03T, subclone D), whilst we detected 23 small cell populations, i.e., subclones less than ten cells (Figure 1A and Supplementary Table S2). We calculated Gini index for each subclone, a higher Gini index reflects greater statistical dispersion of CN among genomic regions in that subclone. Gini indices vary between subclones, illustrating intratumor heterogeneity in LUAD, LUSC, and SCLC (Figure 1B and Supplementary Table S2). Despite intra- and inter- tumor heterogeneities, hierarchical clustering of the subclone CNV profiles showed that some subclones across different patients shared similar CNV profiles at large. We elaborated the similarity in the Supplementary Fig. S4, S5, and the Supplementary Results.

## Punctuated copy number evolution of lung cancer tumors

To investigate the process of copy number evolution, we built a cell-level phylogeny tree and a subclone-level evolutionary tree of lung tumors with their CNV profiles (Supplementary Fig. S6-S8). Together with the consensus CNV profiles at subclone level, we manually infer copy number evolutionary trees (Figure 2A) and observed that almost all lung tumors undergo a punctuated copy number evolution (PCNE) event, that is, the CNVs of subclone are acquired in short bursts of crisis. Then, some subclone may be followed by a branching copy number evolution (BCNE) process, which diverges to more sub-subclones of tumor cells with CNVs intermediate accumulated over evolution time. We hypothesize that copy number evolution begins in normal tissue then diverges into a tumor subclone dominated by amplified (A), diploid (D), or lost (L) copy numbers. The subclone carrying gain, neutral, or loss may also be involved in subsequent evolution; therefore, in each tumor sample, we suffixed an index number to the subclone name to indicate when a subclone occurred during the evolution process (e.g., subclone A2 occurs after subclone A1). We found evidence of punctuated copy number evolution (PCNE) in lung tumors through the cell-level phylogeny trees, where two to five subclones derived directly from a common ancestor. Exceptions happen in LUAD02T and LUSC01T, which populate one dominant cell group with minor clones of less than ten cells. Likewise, four lung tumors (LUAD06T, LUAD07T, LUSC07T, and SCLC01T) show evidence of branching copy number evolution (BCNE) derives subsequent subclones (≥ 10 cells) after PCNE. For instance, Supplementary Fig. S6 depicts LUAD03T undergoing a PCNE event to subclones D, L, LX, and A; then A may evolve to AL with one cell. The common ancestor of LUAD04T evolves subclones D, A1, and LX in a PCNE process. LUSC04T goes through a PCNE process that produces subclones LX and A1, then A1 may evolve to a minor subclone A2 with eight cells. Subclones A, LX, and L1 were derived from a PCNE event in LUSC05T. In SCLC01T, PCNE generates subclones LX and A1, followed by a BCNE process where A1 derives subclones A2. Subclone L with six cells was also detected. Likewise, the PCNE model leads to subclones A, LX, and L1, then L1 can derive to L2 with three cells in SCLC02T. The evolutionary process of the copy number for additional LUAD and LUSC is described in Supplementary Fig. S7-S8, respectively. Moreover, Subclone A of SCLC02T is characterized by the amplification of *MYC* and *ASCL1*, while *MYCN* is amplified in subclone L. Both *MYC* and *MYCN* have been reported to promote SCLC in mice ^27^, the different amplified genes of subclone A and L indicates that these two subclones have different evolution process. *APC* is specifically amplified in A2 subclone of LUAD06T and A2 subclone of LUAD07T. The amplification of *APC* is significantly related to better progression-free survival of TCGA-LUAD (Figure 2B). *CEP89* and *FAT3* which has a high amplification frequency in both LUAD and LUSC tumors also have prognostic significance in TCGA-LUAD (Figure 2C-D).

## Most structural aberrations may occur before or after the punctuated copy number evolution.

Structural aberrations in the genome, such as small insertions and deletions (InDels) and structure variations (SV) in lung cancer, play a critical role in lung cancer progression^24^. High throughput sequencing data analysis suggests that instead of independent occurrences, a group of SVs may arise jointly, which leads to complex rearrangements or complex structure variations (cSV)^28^. Complex structure variations, specially chromothripsis, may cause the landmark fusion oncogenes or the overexpression of oncogenes, thus promoting the tumor progression in LUAD, LUSC, and SCLC^24,29–31^. We have annotated the InDels, SVs, and cSVs of the sixteen lung tumors at subclone-level. As shown in Figure 4A, both LUAD and LUSC have highly mutated samples (LUAD03T, LUAD04T, LUSC04T, and LUSC05T) and relatively low mutation samples, including LUAD05T, LUAD06T, and LUSC02T. Subclone-level deletion (DEL-ht) is prevailing against other SV types across the cohort (Supplementary Table S4).

Interestingly, many genetic alterations (10~70%) co-exist in tumor subclones formed by PCNE throughout the cohort (Figure 3B and Supplementary Table S5). In LUAD03T, the most genetic alterations coexist in PCNE-formatted subclones (28.58% for D, L, LX and A; 40.81% for D, L and LX). In LUAD04T, subclones D, A1, and LX (formed by PCNE) share 57.34% genetic alterations, subclone groups D and LX have 40.14% common genetic alterations. In LUSC04T, the PCNE yielded subclones LX and A1 have 78.10% common genetic alterations, so does the minor subclone A2 (eight cells, 12.03% for A1, A2 and LX). In LUSC05T, 61.15% of the genetic alterations were observed recurrently in subclones A, LX, and L1 derived from PCNE. Subclone groups L1 and LX share 23.68% common alterations as well. Also, in SCLC01T, PCNE derived LX and A1, and BCNE derived A2 and A3 also share a substantial amount of genetic alterations, that is, 10.93% for A1 and LX; 15.60% for A1, A3, and LX; 14.18% for A1, A2, and LX; 31.92% for A1, A2, and A3. The minor subclone L with six cells shares several genetic alterations with other four subclones as well. So do the remaining tumors (Supplementary Fig. S9-10). These aforementioned findings indicate that most genome aberrations may occur before or after the CNV burst. We constructed the single-cell phylogeny tree based on CNV (amplified, neutral, or deleted) and SV (occurred or not) to validate this speculation. The tree inferred from SV plus CNV is concordant with the CNV derived subclone structure, indicating that a large proportion of genome variations may happen before the PCNE event. (Supplementary Fig. S11-13). Moreover, we observe the intra-tumor genetic alteration diversity across the cohort. Several tumor subclones harbor exclusive InDels, SVs, and/or cSVs, consistent with the hypothesis of a BCNE process after a punctuated burst of CNV.

Breakage-fusion-breakage (BFB) is a complex rearrangement that results in stair-like copy number amplifications that may occur accompanying the punctuated evolution events. We detected two BFB events in our lung tumor samples. In LUSC08T (Supplementary Fig. S14), the breakpoints of four fold-back inversions (FBIs) split the local genome region of chr1 in subclone A1 into seven segments. Based on the CNV of subclone A1, we inferred the rearrangement structure with four BFB cycles. With one FBI missing in subclone A3, the reconstructed BFB structure, like that of subclone A1, is derived from four BFB cycles. However, subclone A4 only contains one FBI, the reconstructed BFB structure is different from subclone A1 and A3, derived from two BFB cycles. The heterogeneity of BFB structure in subclone A4 indicates the branching evolution happened after the punctuated occurrence of PCNE. The BFB structure in three subclones all duplicate the oncogenes PLA2G4A that may drive the tumor malignancy. As for SCLC01T (Supplementary Fig. S15), the breakpoints of three FBIs split the local genome region of chr3 in subclone A1 into seven segments. We inferred the rearrangement structure from four BFB cycles. With one FBI missing in subclone A2, the reconstructed BFB structure, like that of subclone A1, is derived from four BFB cycles. This BFB event amplify oncogene GBE1 that could foster the aggressiveness of tumor.

Despite the intra- and inter- tumor heterogeneities, we identified several genetic alterations that occurred recurrently in cancer-related genes in LUAD and/or LUSC, we illustrate and discuss them in Figure 4C and Supplementary Results.

## Complex rearrangements before or upon PCNE recurrently hit MHC II genes on LUSC tumors on subclone-level

MHC genes play an important role in immune response of cancer^32^. We found that the InDel (small insertion and deletion), SV (DEL-ht, TRX-ht, DUP-th, TRX-th, INV-hh, TRX-hh, INV-tt, and TRX-tt), and cSV (Chromothripsis and other cSV) recurrently hit HLA genes in each tumor subclone (Figure 3C and Supplementary Fig. S16A). We detected complex rearrangements harboring in one group of MHC II genes. A group of SVs disrupted HLA-DRB5, HLA-DRB6 and HLA-DRB1 on 2/7 LUAD samples, 6/8 LUSC samples, and 1/2 samples (Figure 3C and Supplementary Fig. S16B-C). The occurrence of SV breakpoints in the HLA-DRB gene family for LUSC is high. We found four hotspot (occurred larger than three times) SV breakpoints for HLA-DRB5 (chr6:32,522,945, chr6:32,523,063, chr6:32,523,983, and chr6: 32,528,226) and two for HLA-DRB6 (chr6:32,558,989 and chr6:32,559,177). These complex rearrangements exhibit a similar pattern by consisting of alternating deletions (DEL-ht) and duplications (DEL-th). Then we check the subclone-level occurrence of the corresponding SVs (Supplementary Fig. S17-19). These SV events almost appear at every major subclone of tumors yielded by PCNE. These findings further suggest that complex rearrangements in MHC II genes may occur prior to or upon CNV burst. These structure variations cut and paste MHC II genes into pieces, leading to a possible malfunction of the immune response. Furthermore, the MHC-associated cSV occurs more frequently in LUSC than LUAD, consistent with the fact that LUAD achieves a better response in immune therapy. The single cell transcriptome analysis of an independent NSCLC case-control cohort is also consistent with the aforementioned findings. In Supplementary Fig. S20, the malignant cells from NSCLC showed that HLA-DRB5 highly expressed in AT2 (Type II alveolar) and club cells of LUAD and in fibroblasts and myeloid cells of normal tissues, and HLA-DRB1 in fibroblasts of LUAD. While in LUSC, both HLA-DRB5 and HLA-DRB1 are only highly expressed in normal tissue, and HLA-DRB5 mainly in neutrophils, B, and natural killer cells; HLA-DRB1 in Basal and B cells.

## Similar and recurrent copy number and structure variation substructures across different patients for lung tumor samples

Despite intra- and inter- tumor heterogeneities, hierarchical clustering of the aggregated subclone CNV profiles showed that some subclones across different patients could share similar CNV profiles at large (Supplementary Figure S4A). A cluster of diploid subclones shows extremely low Gini coefficients (Figure 1C and Supplementary Figure S4A). Single cells in these diploid subclones share a consistent neutral CNV profile (CN 2). We observed a large group of subclones “LX” from female patients kept most of the genomic region diploid while exposing the loss of heterozygosity (LOH) on almost the whole chromosome X. Moreover, LUAD, LUSC, and SCLC all exhibit subclones with LOH or CNV gains across the whole genome. A group of LUSC subclones tends to be more similar with dominant copy number gains (LUAC01 A, LUSC04 A1, LUSC08 A2, LUSC08 A3, and LUSC07 A1), as evidenced in hierarchical clustering and UMAP plots (Supplementary Figure S4). We then fitted subclone-level consensus CNV profiles into GISTIC2.0 [[1]](#_bookmark21) to check the recurrent CNVs in lung cancer histology. We detected subclone-level focal amplifications and deletions in LUAD, LUSC, and SCLC (Supplementary Figure S5A-B). The three lung cancer subtypes had distinct chromatin distribution characteristics on CNV gains. Specifically, LUAD harbored tumorigenesis-promoted TP53, SFTP, and GABR families; LUSC mainly contained keratinocyte-development-associated genes S100A and SPRR family, FCGR family, and many immune or inflammatory factors (CD1A, CD48, IL6R, etc.). The SCLC harbors recurrent gains on CST and DEFB family members. GO analysis of focally amplified genes also showed that LUSC enriched in the immunoglobulin, IgG, and RAGE receptor binding, while LUAD participated in GABA-gated chloride ion channel and GABA activities, and SCLC in endopeptidase and peptidase-related activities (Supplementary Figure S5C-D). On the focal deletions, LUAD harbored a loss on SKT11, while LUSC on SERPINB family genes and overexpression of SERPINB genes suppressed the invasiveness and motility of malignant cancer cells [[3].](#_bookmark23) KEGG and GO analysis revealed that both LUAD and LUSC applied the olfactory transduction pathway, which are putative drivers of cancer [[4].](#_bookmark24) In contrast, LUAD specifically participated in VEGF and GnRH signaling and Spliceosome pathways, and LUSC in the Butanoate metabolism pathway (Supplementary Figure S5C-D). Thus, LUAD and LUSC share more similarities in the current loss compared to SCLC.

Despite the intra- and inter- tumor heterogeneities, we identified several genetic alterations that recurrently occurred in cancer-related genes in LUAD and/or LUSC (Figure 3C and Supplementary Table S6). NSCLC oncogenes ALK, GNG2, RARB, and RXRA are recurrently mutated in both LUAD and LUSC. B4GALT4, CHSY1, and MAPR4 are found exclusively recurrently mutated in LUAD. Several LUSC distinctive mutant genes were obtained, including GLI2 (oncogene), PIK3R3 (PI3K-Akt signaling pathway), and genes belonging to metabolic pathway (ATP6V0E2, CHPT1, GALNT14, and NDUFA12). Likewise, we detected several complex rearrangements in LUAD, LUSC, and SCLC (Figure 3). There is cancer related-genes associated with complex rearrangements that occur on both LUAD and LUSC (Figure 3C), including CEACAM3, CEP85L, CHSY1, CNTNAP5, GSTTP2, GPHN, MLIP, MUC3A, SKA3, and SUGCT. Furthermore, we observed cSV-linked DDX10 and SKA3. There are some LUAD distinct cSV-related genes, including DEPDC1, RAF1, MUC19, etc. In terms of LUSC, we found cSV hit oncogene SPOCK3. We detected complex rearrangement associated CCDC32-CBX3 together in LUSC04T and TDG-TMEM132B in two LUSC samples.

We found that some subclones across different patients can share similar CNV and SV profiles at large and copy number gains and losses recurred in LUAD, LUSC, and SCLC.

# Supplementary Discussion

Current lung cancer single-cell genomics applications were virtually limited to circulating tumor cells (CTC) in small-cell lung cancer (SCLC)^21–23^. The intra-tumor heterogeneity lies on the primary solid NSCLC nodules in single-cell resolution remain unsolved; the single-cell intra-tumor heterogeneity reflects on structure variations and complex rearrangements remain unclear for their contribution to the tumor progress in LUAD, LUSC, and SCLC. In this study, we applied the 10x single-cell CNV solution to sixteen lung cancer tumors, including seven LUAD, seven LUSC, and two SCLC, and comprehensively investigated the copy number and structure variation subclone evolution in solid lung cancer with a single-cell resolution.

In total, we harvested 13,343 non-noisy single cells and obtained 16 to 33 cell clusters, which were further annotated into 3 to 9 distinct subclones in each tumor, with 72 subclones in total. Our results indicate that lung cancer tumors have huge subclone diversity in copy number variations and complex structural variations, whilst we also found similar and recurrent copy number substructures across patients. Clonal analysis indicates lung tumors undergo a punctuated copy number evolution (PCNE) event, followed by a branching evolution (BCNE) process. We detected two breakage-fusion-bridge events in lung squamous cell carcinomas that duplicate the oncogenes PLA2G4A and GBE1 separately may occur accompanying the punctuated evolution events. We found complex rearrangements harbor in two major histocompatibility complex (MHC) class-II protein-encoding genes (HLA-DRB5 and HLA-DRB1) on lung cancers, especially with high frequency (75%) in squamous tumors. Further evolutionary analysis suggests these complex rearrangements may occur prior to or upon the PCNE process. Hence, our results reflect lung tumors holding huge subclone diversity on copy number variations and complex structure variations.

Our data demonstrates that all LUAD, LUSC, and SCLC tumors are polyclonal, the copy number substructure in each lung cancer tumor consists of an array of cell clusters (16–33) which can be further annotated into a few distinct subclones (3–9). The number of cell clusters and subclones is consistent with several scDNA-Seq studies of breast cancer^9,18–20^. Beyond the clonal diversity intra- and inter- tumors, we observed the CNV conservativeness preserved across LUAD and LUSC tumors. Our analysis also showed that LUAD mainly harbored gains in tumorigenesis-promoted gene families, such as TP53, SFTP, and GABR. While LUSC mainly harbored gains in immune related gene families (S100A, SPRR, and FCGR) and factors (CD1A, CD48, IL6R, ect.)^33^. SCLC mainly harbored gains in CST and DEFB family members. For focal deletions, the SKT11 deletion mutation was found in LUAD, which was associated with the absence of PD-L1 and could directly induce resistance to immunotherapy. LUSC has focal deletions in genes in the SERPINB family related to the invasiveness and motility of malignant cancer cells^34^. However, except for the distinct features of the tumor subtype, LUAD and LUSC all have focal lost genes in the olfactory transduction pathway, which are admitted as putative drivers of cancer^35^. These results illustrate the distinct chromatin distribution characteristics on subclone-level CNV gains in LUAD, LUSC, and SCLC and more similarities on current loss in LUAD and LUSC compared to SCLC.

Our study reports that punctuated copy number evolution (PCNE) is prevailing among LUAD, LUSC, and SCLC, with extensive genomics gains and LOH are significant early evolutionary events that may burst in a short time. These findings are consistent with a series of breast cancers of single-cell genomic study which reported PCNE^9,18–20^. Several tumor data further exhibit evidence of branching copy number evolution (BCNE) after PCNE. The results are consistent with the evolution model proposed by Minussi et al. where the upcoming transient instability produces extra subclones after PCNE^20^. Our analysis suggests that the tumor cells continue to explore the fitness landscape during the growth and expansion of the solid lung cancer tumor.

We reported the huge diversity of subclone-level structure variations and complex rearrangements in lung cancer tumors. Our results represent an arsenal of cancer-related genes that are common or distinct among lung cancer histology subtypes. The famous oncogenes ALK, GNG2, RARB, and RXRA of NSCLC are mutated recurrently in both LUAD and LUSC. B4GALT4, CHSY1, MAPR4 are found to be exclusively recurrently mutated in LUAD. Several LUSC distinctive mutant genes were obtained, including GLI2 (oncogene), PIK3R3 (PI3K-Akt signalling pathway), and genes belongs to metabolic pathway (ATP6V0E2, CHPT1, GALNT14 and NDUFA12). Our analysis demonstrates that LUAD and LUSC all exhibit a number of complex rearrangements, whilst the occurrence of chromothripsis is higher in LUSC and SCLC than in LUAD. Our data also exhibited some cancer related-genes, which were involved in the cSV regions in both LUAD and LUSC (Figure 3C), including CEACAM3 (tumor biomarker), CEP85L (a breast cancer antigen), CHSY1 (tumor-promoting^36^), GSTTP2 (DDTL, GSTTP2, fusion gene^37^), GPHN (LUSC suppressor^38^), MLIP, MUC3A (induces PD-L1 in NSCLC^39^), SKA3 (EGFR–PI3K–Akt^40^), and SUGCT, and genomic structure aberrations including cSV on both smokers (LUAD02T, LUAD06T, LUSC05T) and non-smokers (LUAD04T and LUSC07T). Such as the cSV linked DDX10 and SKA3, which has reported recurrent fusion between them in breast cancer^41^ and DDX10 could as a novel tumor suppressor^42^. There exists some LUAD distinct cSV related genes, containing DEPDC1B (enhances migration and invasion of NSCLC cells^43^), RAF (RAS/RAF/MEK/ERK and regulates cell migration^44^), MED12L (play role in actin-mediated cytokinesis via LIMK2/cofilin pathway in NSCLC^45^), MUC19 mutations (good responses to anti-PD-1 inhibitors in NSCLC^46^) and PRKRA (therapeutic target in MOC^47^). In terms of LUSC, cSV related SPOCK3 may be the potential biomarker in prostate cancer^48^, and some sample specific complex rearrangement were detected, including CCDC32-CBX3 in LUSC04T and TDG-TMEM132B in two LUSC samples. The fusion of the CCDC32-CBX3 gene was recurrent (10%) in a cohort of neuroblastomas48, and TMEM132B was frequently mutated in small cell lung cancer and pancreatic cancer, TDG rs4135050 (A/T) could be an early diagnosis marker of diseases caused by smoking. Therefore, the appearance of complex rearrangements and aberrations in genomic structure in genes may lead to tumor progression, and LUSC shows more complex changes in gene structure.

We found that InDels and SVs prevail in major histocompatibility complex (MHC) genes. Our data demonstrate complex rearrangements harbor in two protein encoding MHC II genes (HLA-DRB5 and HLA-DRB1) regardless of lung cancer histology types, and specially in LUSC with high frequency (75%). Evolutionary analysis suggests these complex rearrangements may occur prior to or upon the PCNE process. MHC molecules are usually only present in specialized antigen-presenting cells such as dendritic cells, mononuclear phagocytes, some endothelial cells, thymic epithelial cells, and B cells, which play an essential role in immune responses. The complex rearrangement event disrupted HLA-DRB5 and HLA-DRB1 and resembled them in a wrong order, thus leading to a potential malfunction of the immune response. Furthermore, MHC-associated cSV occurs more frequently in LUSC than in LUAD, consistent with the fact that LUAD achieves a better response in immune therapy. Single-cell transcriptome analysis on an independent NSCLC case-control cohort also shows that the malignant cells from NSCLC also revealed that HLA-DRB5 and HLA-DRB1 are all mainly highly expressed in immune cells of LUSC compared to LUAD. These data serve as an important indication for the lung cancer immune-therapy.

One of the restrictions placed on our study is that the detected number of subclones hinges on the number of cells profiled by a 10x single cell CNV solution, unable to present the whole panorama of subclone diversity in lung tumors, but this is a common problem in the sequencing study design^20^. Next, the 10x protocol enables scalable whole genome copy number profiling while the average coverage of each single cell is relatively low (~0.03X). Thus, the SV and cSV in some minor subclones may be concealed. We have compared the subclone-level copy number and structural variation landscape between LUAD and LUSC; however, our results were unable to derive a statistically significant difference due to the limited number of tumors. We plan to collect and sequence more lung tumors to enrich our findings.

In total, we sequenced 13,343 non-noisy single cells from LUAD, LUSC, and SCLC. Our results indicate that lung cancer tumors have huge subclone diversity in copy number variations and complex structural variations, whilst we also found similar and recurrent copy number substructures across patients. Clonal analysis indicates lung tumors undergo a PCNE event, followed by a BCNE process. We detected two breakage-fusion-bridge events in lung squamous cell carcinomas that duplicate the oncogenes PLA2G4A and GBE1 separately may occur accompanying the punctuated evolution events. We found complex rearrangements harbor in two major histocompatibility complex (MHC) class-II protein-encoding genes (HLA-DRB5 and HLA-DRB1) on lung cancers, especially with high frequency (75%) in squamous tumors. Further evolutionary analysis suggests these complex rearrangements may occur prior to or upon the PCNE process. Hence, our results reflect lung tumors holding huge subclone diversity on copy number variations and complex structure variations.

# Supplementary Methods

## Sample collection

Our study was approved by the Institutional Review Board of West China Hospital and informed consent was obtained from each patient. Fresh tumors were obtained from patients with primary lung cancer at West China Hospital. Disease stage was determined by the 8th edition of the American Joint Committee on Cancer (AJCC) TNM stage system^49^. Clinical characteristics of these samples were recorded at recruitment listed in Supplementary Table S1.

The freshly obtained tissues were cut into small pieces on ice, and digested with collagenase I/IV in HBSS at 37 °C for 30 minutes. The digested tissues were filtered through a 40 μm nylon mesh and the filtered cells were incubated with red blood cell lysate for 5 minutes. After centrifugation, cells were washed once with HBSS and suspended in 0.04% BSA in PBS. The cell suspensions were directly processed for single-cell DNA-Seq according to the manufacturer's instructions. Alternatively, cell suspensions were frozen in cell cryopreservation solution at -80°C, and used for single-cell DNA-Seq after thawing.

## 10x CNV sequencing

Chromium single cell DNA Library kits (10X Genomics, Pleasanton, CA, United States) were used to generate single-cell DNA libraries according to the manufacturer's instructions. The sequencing libraries were then sequenced on the Illumina NovaSeq 6000 platform using paired end reads of S4 chemistry 2 × 150 (300 cycles) paired-end reads.

## CNV analysis

The copy number profiles of single cells were obtained using the official 10x CNV solution tools cellranger-dna with default settings^11^. The cell summary statistics are stored in Supporting Tables. Noisy cells were removed in the downstream analysis. We downloaded the default cut dendrogram built by hierarchical clustering from 10x CNV visualization tool Loupe^11^ (Supplementary Figures S1-S3) The tumor cell groups were obtained from cut dendrogram. We assigned cells grouped by a leaf node in the cut dendrogram as cell clusters; cells inside one cell cluster sharing similar CNV profiles. Then, we manually merged cell clusters into major tumor subclones based on the similarity of their CNV profile (Supplementary Figures S1-S3). Here, a subclone consists of either cell clusters that share a subset of CNV events or a distinct cell subpopulation with a CNV profile that differs from other clusters. We calculated the subclone-level consensus CNV profiles by averaging the CNVs of all cells in that subclone. Then, we fitted subclone-level consensus CNV profiles into GISTIC2.0^50^ to check the recurrent CNVs in LUAD, LUSC, and SCLC, respectively.

## SV analysis

We call the genetic alterations (InDels and SVs) by fitting bulk BAM into SvABA^51^, and filter alterations with “PASS”. Imprecise structure variations without the support of split reads are also filtered. The InDels and SVs were annotated using AnnotSV^52^. Complex structure variations and chromothripsis analysis and visualization were performed with scSVAS with default settings. Generally, SV events with breakpoints less than 1,000 base pairs were grouped together as a complex structure variation event. We identified complex structure variation event chromothripsis following the criterion proposed by Korbel and Campbell^53^. We detected BFB with Ambigram (https://github.com/deepomicslab/Ambigram).

## Evolutionary analysis and interpretation

We built the cell-level phylogeny tree and subclone-level lineage tree of lung tumors with their CNV profiles by FastTree^54^ with default settings. Amplified, neutral, and lost CNV bin was converted into nucleotides “A”, “C”, and “G”, respectively. For CNV plus SV phylogeny tree, we convert the occurrence of one SV event to nucleotide (occurred: “A”; otherwise: “C”). We visualized the phylogeny tree with the R package “ggtree”^55^.

Punctuated copy number evolution (PCNE) hypothesizes the CNVs of each subclone are acquired in short bursts of crisis, while branching copy number evolution (BCNE) hypothesizes the CNVs of subclones are intermediate accumulated over evolution time ^9,18–20^. In this study, we assume that the punctuated evolution has two patterns as described by Navin et al. ^9,18–20^: 1) there are significant differences in the consensus CNV profiles of subclones, and 2) the subclone branches appear at the early stages of the cell-level phylogeny trees. We assume that branching evolution following punctuated evolution will result in two patterns as described by Minussi et al. ^9,18–20^: 1) cumulative copy number losses or gains in child subclones compared to the parent subclones, and 2) along a branch of the phylogeny tree, the child subclones appear after their parent nodes. To interpret the possible PCNE or BCNE process on the lung tumor subclones, we firstly category each sample subclone with “P” (punctuated), “B” (branching), or “NA” labels with two steps (Supplementary Table S2): 1) we labeled subclones whose single cell numbers less than ten as “NA” since the size of subclone is too small to draw an evolution hypothesis; 2) we manually assigned “P” and “B” labels to the remaining subclones by observing the punctuated and branching patterns mentioned above. Next, we assigned each tumor sample a label from “PCNE”, “PCNE+BCNE”, “PCNE+Minor BCNE”, and “NA” (Supplementary Table S2). “PCNE” refers to the presence of a tumor sample consisting of a minimum of two subclones, which may be attributed to PCNE. “PCNE+BCNE” indicates the existence of a tumor sample with a minimum of two subclones may be caused by potential PCNE, and at least one subclone exhibiting signs of possible branching evolution. “PCNE+Minor BCNE” signifies the presence of a tumor sample with a minimum of two subclones may be caused by potential PCNE, and at least one subclone with fewer than ten cells displaying possible branching evolution signatures. “NA” indicates that the tumor sample possesses only one subclone with a cell count of ten or more.

## CNV prognostic analysis

To select the key cancer-related genes for each branch, we filtered the subclone-specific focal CNVs obtained by GISTIC2.0 (Amplification: fold change >= 1; Deletion: fold change <= -0.5). Then we labeled each subclone with subclone-specific copy number amplified or decreased genes that belong to the cancer gene census. As these cancer-driving genes can drive cancer development, we also explored the prognostic significance of these genes with TCGA dataset. We used the R package “RTCGAToolbox” to retain the GISTIC CNV data and clinical data of TCGA-LUAD and TCGA-LUSC. Then we used the R package “survminer” to plot the survival curves for selected key cancer-related genes.

# Supplementary Figures


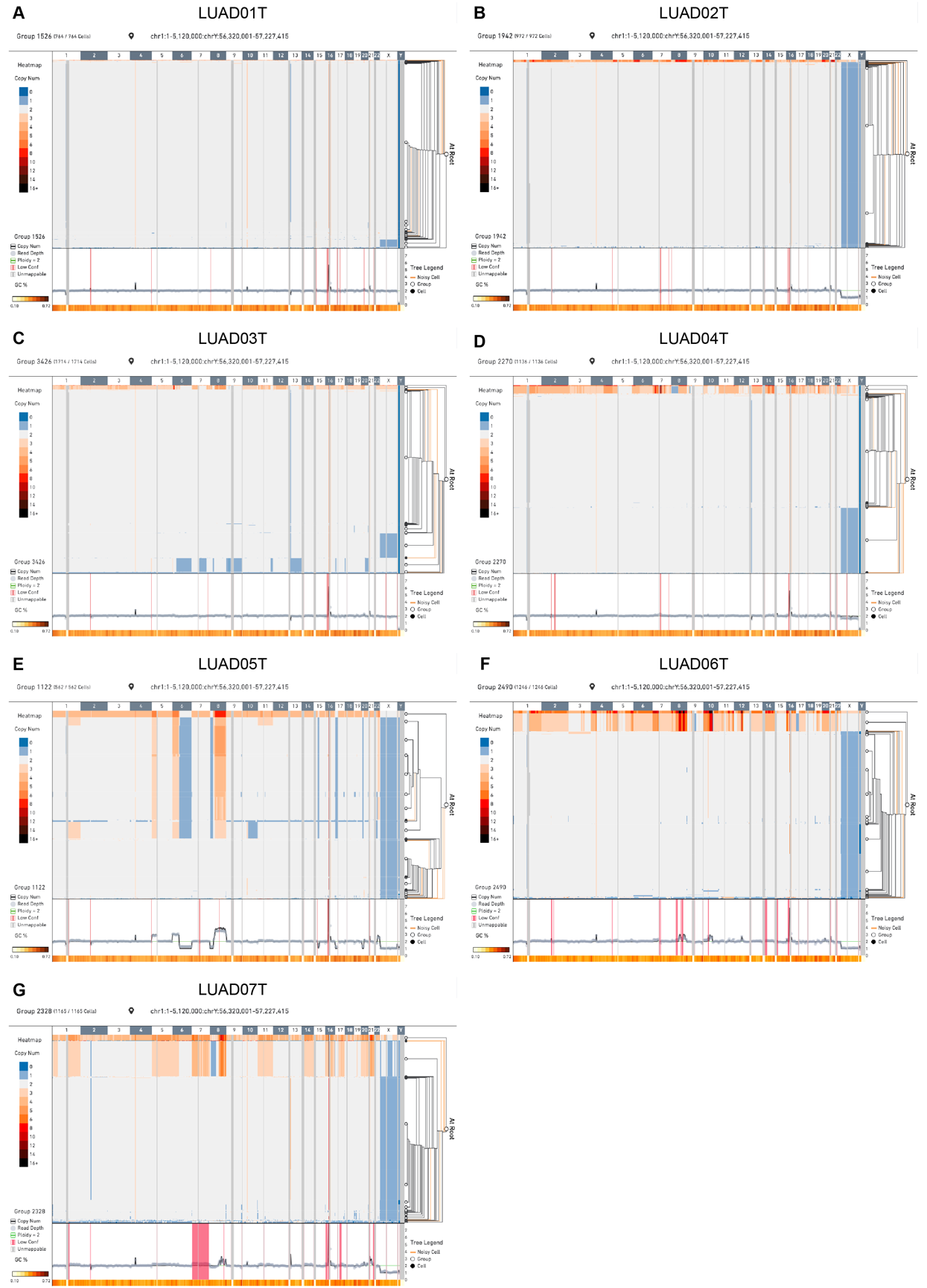


## Figure S1: Copy number substructure of seven LUAD tumors.

(A-G) The heatmap of copy number profiles with single cells as rows and genomics bins as columns for LUAD01T-LUAD07T. The blue, white, and red color in the heatmap represents copy number lost, neutral, and gain, respectively. The grey tile denotes the missing copy number in the corresponding genomic region. The right panel is the cut dendrogram generated by hierarchical clustering. The orange line refers to the noisy cell. The bottom panel shows additional information across the genomic region, including mean copy number, read depth, low confidence region, and GC contents. The figures were obtained from 10x Genomics Loupe. CNV: copy number variation. LUAD: lung adenocarcinoma. LUSC: squamous cell cancer. SCLC: small-cell lung cancer.


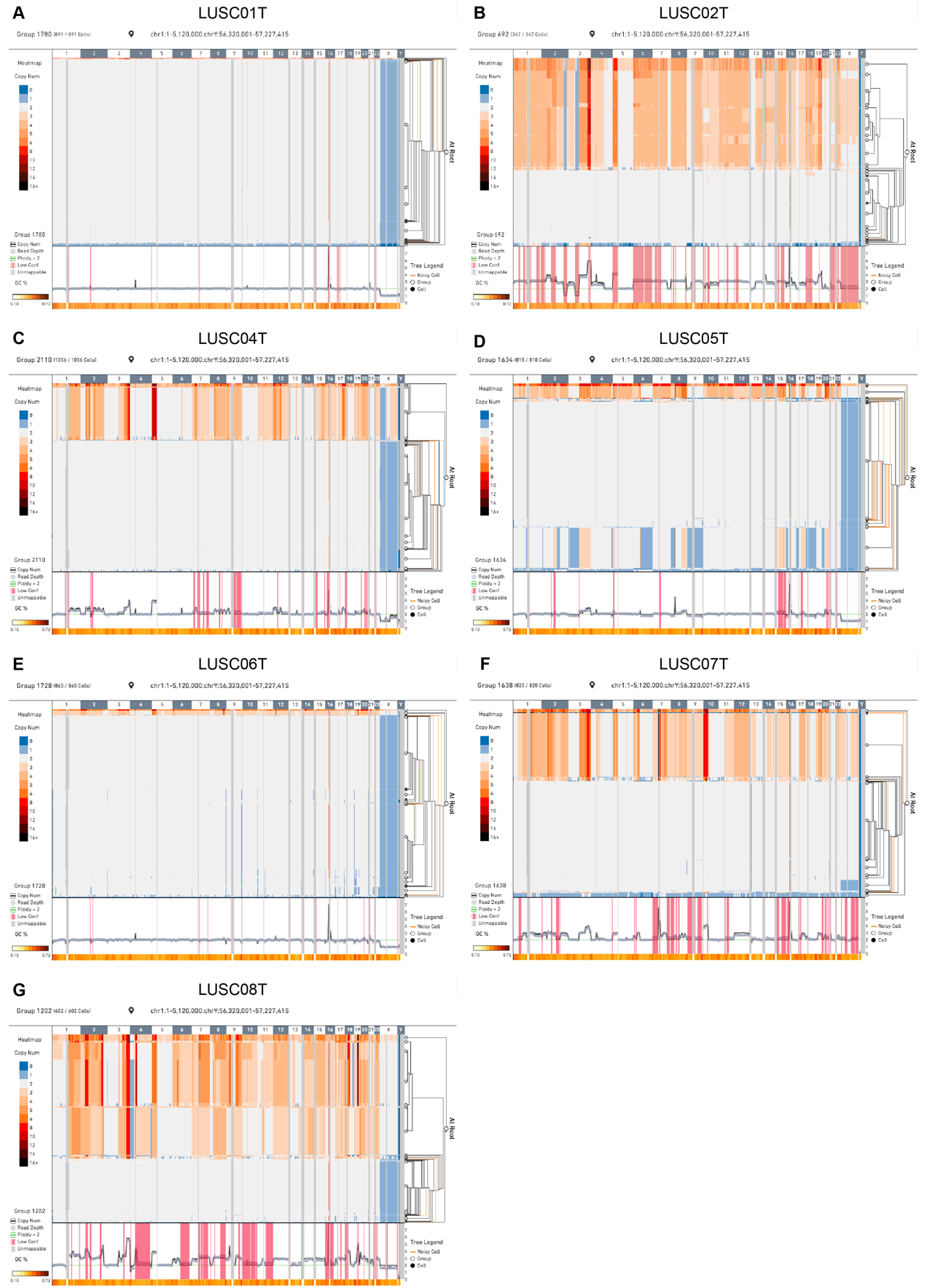


## Figure S2: Copy number substructure of seven LUSC tumors.

(A-G) The heatmap of copy number profiles with single cells as rows and genomics bins as columns for LUSC01T-LUSC02T and LUSC04T-LUSC08T. The blue, white, and red color in the heatmap represents copy number lost, neutral, and gain. Grey tile denotes the missing copy number in the corresponding genomic region. The right panel is the cut dendrogram generated by hierarchical clustering. The orange line refers to the noisy cell. The bottom panel shows additional information across the genomic region, including mean copy number, read depth, low confidence region, and GC contents. The figures were obtained from 10x Genomics Loupe. CNV: copy number variation. LUAD: lung adenocarcinoma. LUSC: squamous cell cancer. SCLC: small-cell lung cancer.


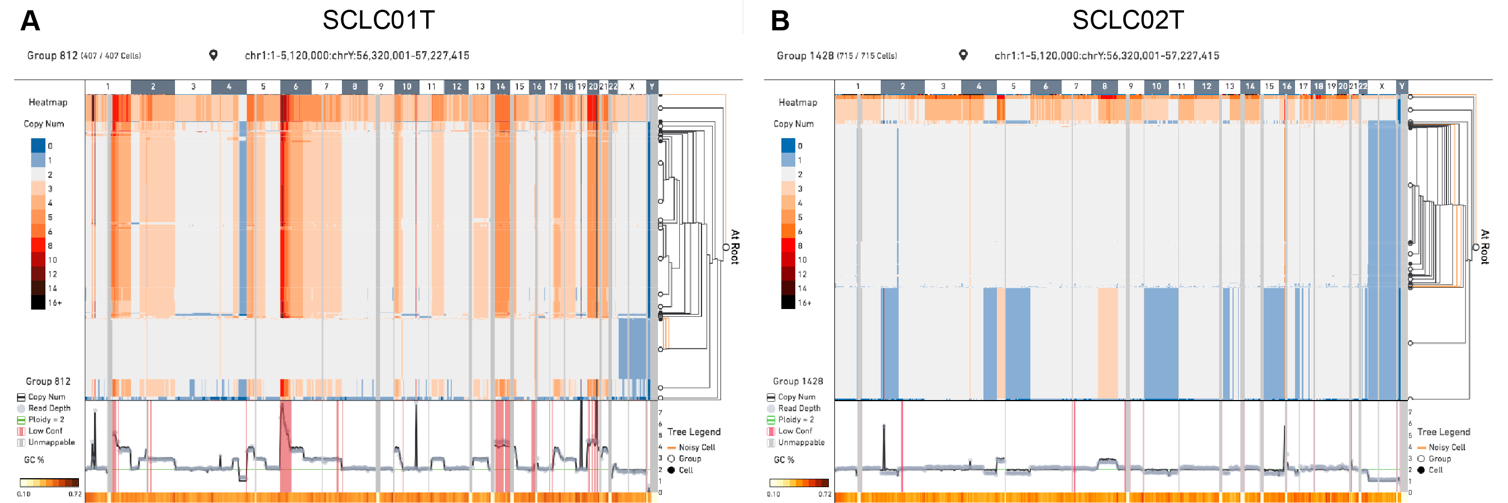


## Figure S3: Copy number substructure of two SCLC tumors.

(A-B) The heatmap of copy number profiles with single cells as rows and genomics bins as columns for SCLC01T-SCLC02T. The blue, white, and red color in the heatmap represents copy number lost, neutral, and gain. Grey tile denotes the missing copy number in the corresponding genomic region. The right panel is the cut dendrogram generated by hierarchical clustering. The orange line refers to the noisy cell. The bottom panel shows additional information across the genomic region, including mean copy number, read depth, low confidence region, and GC contents. The figures were obtained from 10x Genomics Loupe. CNV: copy number variation. UMAP: uniform manifold approximation and projection. LUAD: lung adenocarcinoma. LUSC: squamous cell cancer. SCLC: small-cell lung cancer.


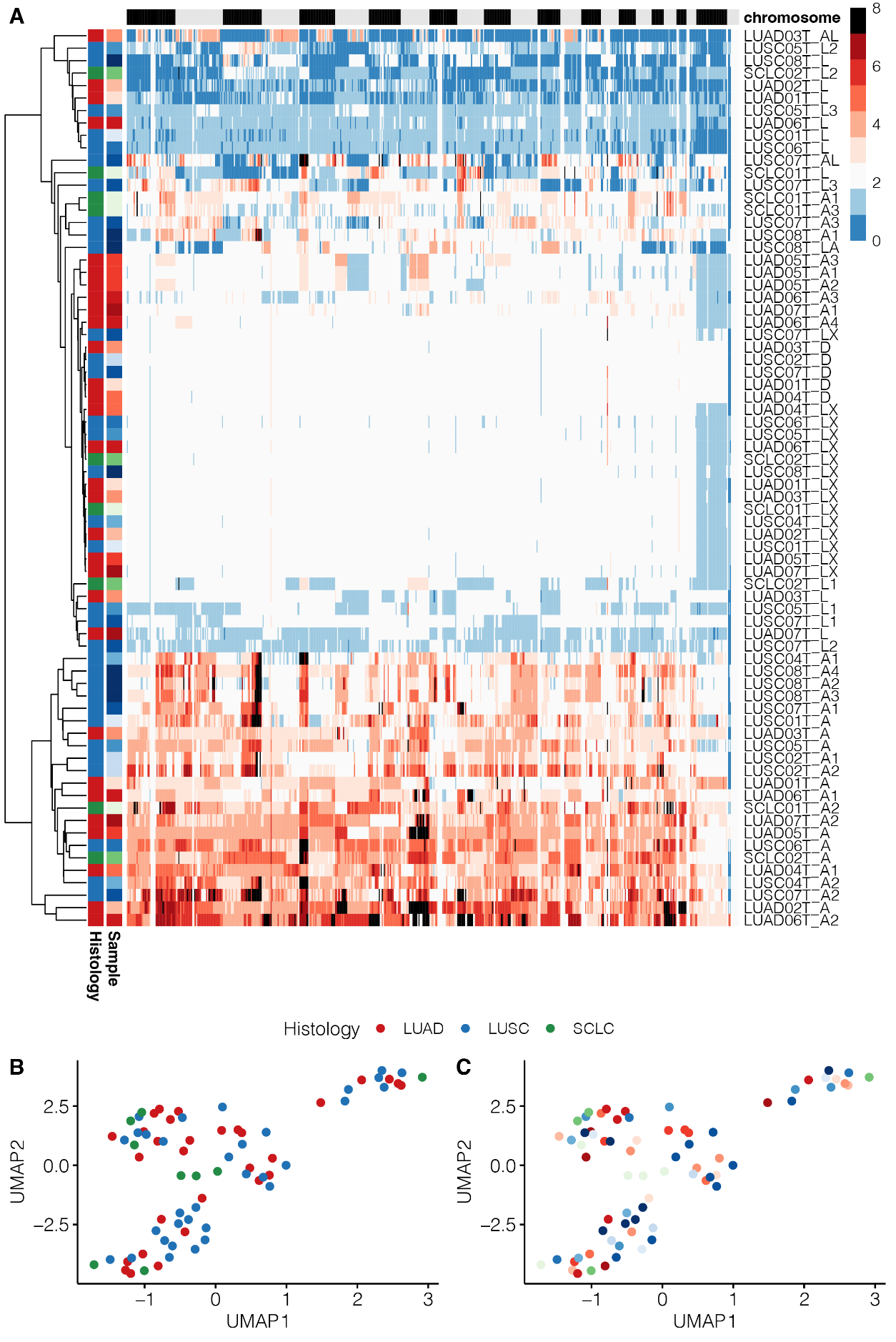


## Figure S4: Subclone-level copy number landscape across the cohort.

(A) Hierarchical clustering plot of subclone-level CNV profiles across the cohort. The blue, white, and red color in the heatmap represents copy number lost, neutral, and gain. (B-C) UMAP plot of subclone-level CNV profiles across the cohort annotated by histology subtype and sample name. Red, blue, and green dots represent LUAD, LUSC, and SCLC subclones, respectively. CNV: copy number variation. UMAP: uniform manifold approximation and projection. LUAD: lung adenocarcinoma. LUSC: squamous cell cancer. SCLC: small-cell lung cancer. N: normal. A: amplification. D: diploid. L: loss. LX: loss on chromosome X.


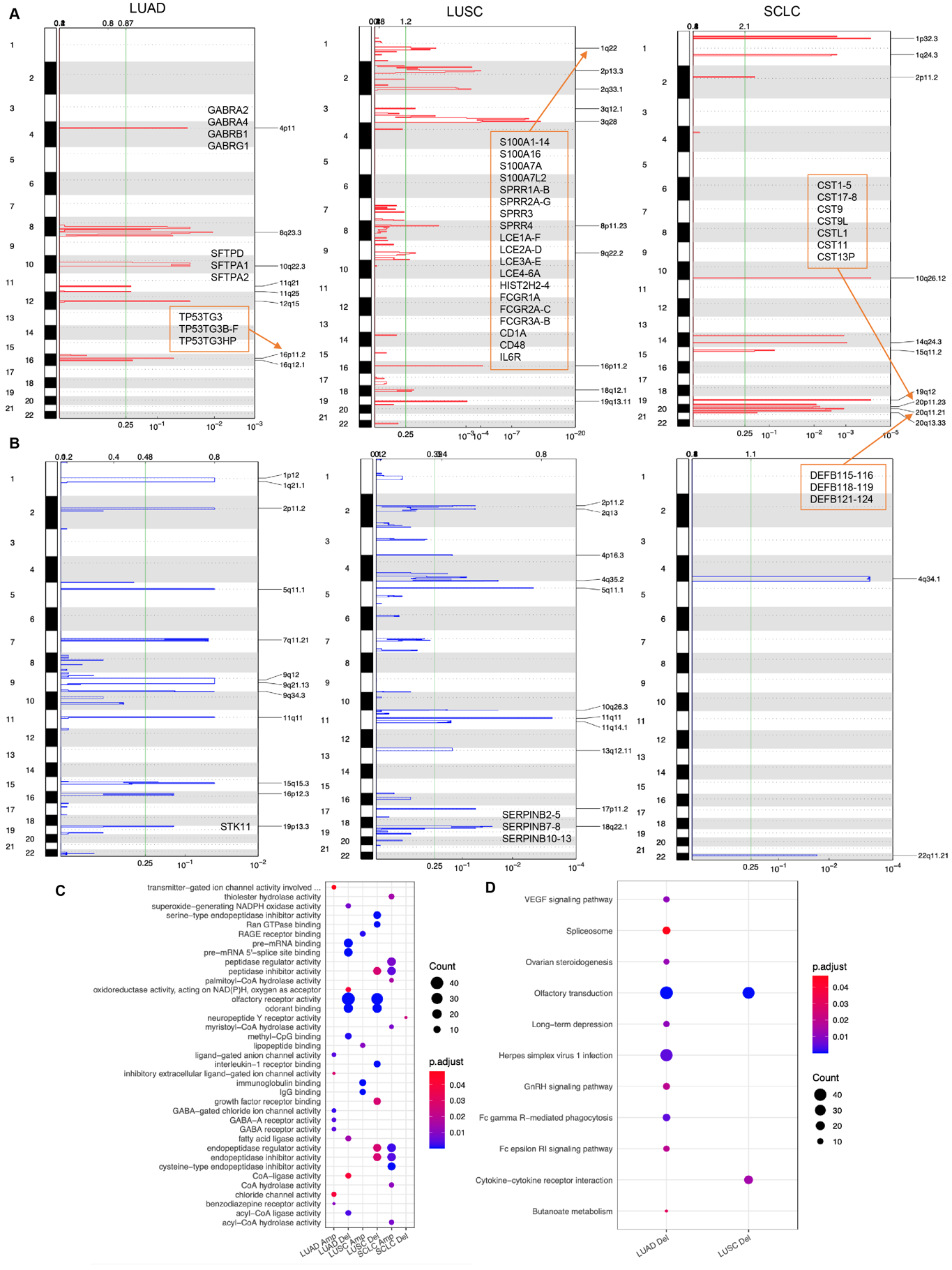


## Figure S5: Subclone-level focal gains and losses across the cohort.

(A) Recurrent amplifications in LUAD, LUSC, and SCLC in GISTIC2.0 analysis. (B) Recurrent deletions in LUAD, LUSC, and SCLC in GISTIC2.0 analysis. (C) KEGG enrichment of focal amplified or lost genes across the cohort. (D) GO enrichment of focal genes across amplified or lost genes across the cohort. LUAD: lung adenocarcinoma. LUSC: squamous cell cancer. SCLC: small-cell lung cancer. KEGG: Kyoto Encyclopedia of Genes and Genomes. GO: Gene Ontology.


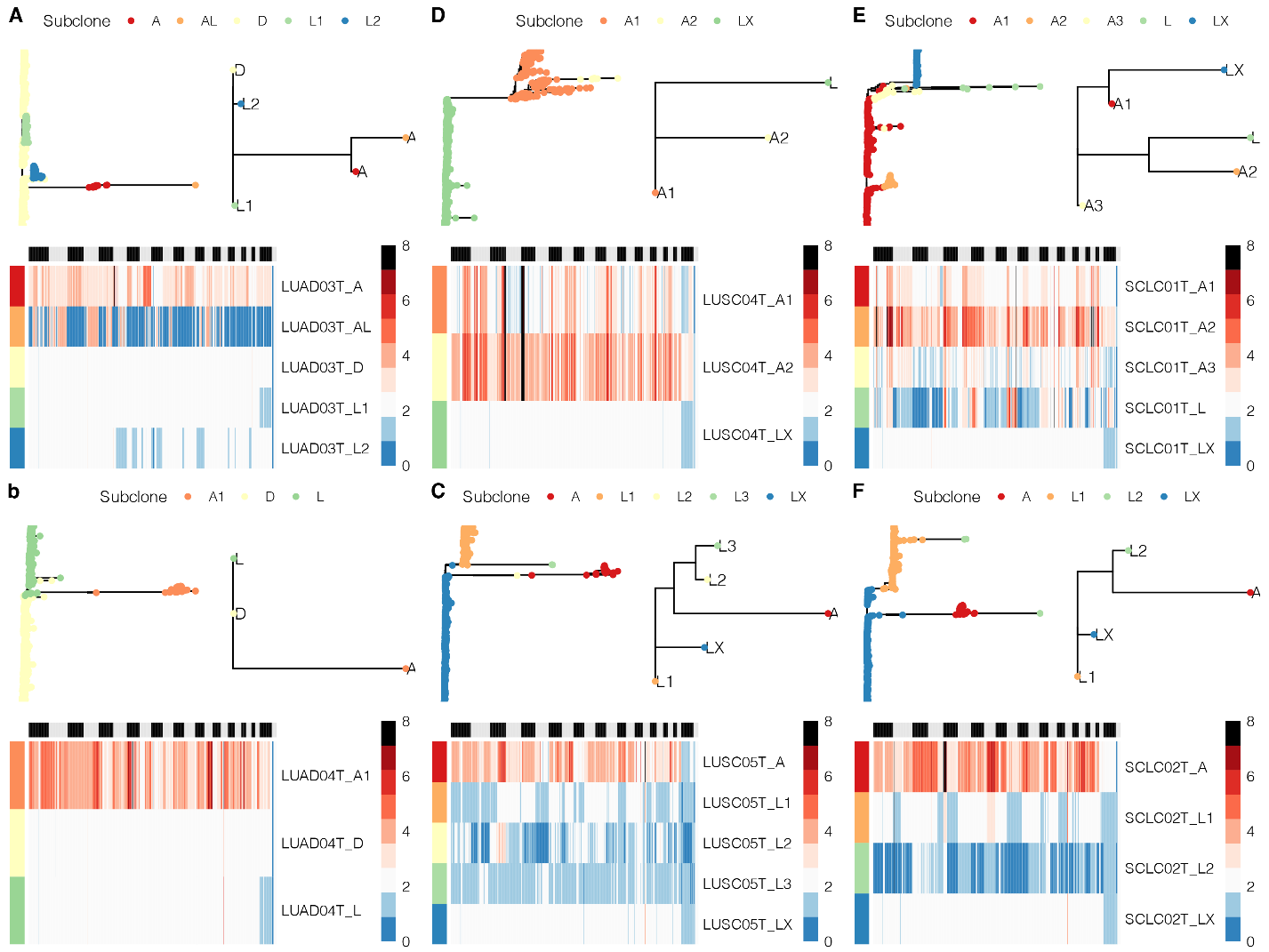


## Figure S6. Copy number evolutionary interpretation on lung cancer tumors.

(A-F) Results of the evolutionary analysis of the copy number for LUAD03T, LUAD04T, LUSC04T, LUSC05T, SCLC01T and SCLC02T. The upper layer comprises the phylogeny tree derived from cell-level CNV profiles and subclone-level consensus CNV profiles. The bottom layer shows the heatmap of the consensus CNV profile at subclone level across the genome. (A) illustrates LUAD03T undergoes a possible PCNE to subclones D, L, LX, and A. Then A may have a branch derive to AL (one cell). (B) shows the common ancestor of LUAD04T evolved subclones D, A1, and LX in a possible PCNE process. (C) demonstrates LUSC04T a possible PCNE process yields subclones LX and A1, then A1 may derive a minor subclone A2 with eight cells in a branch. (D) depicts subclones A, LX, and L1 may be derived from a PCNE event in LUSC05T. Small cell groups L2 (one cell) and L3 (two cells) are further detected in a possible branch. (E) In SCLC01T, PCNE generates subclones LX and A1, followed by a possible BCNE process in which A1 may derive subclones A2 and A3. Subclone L with six cells was also detected. (F) reveals that in SCLC02T the PCNE model may lead to subclones A, LX, and L1, then L1 may derive to L2 with three cells in a branch.


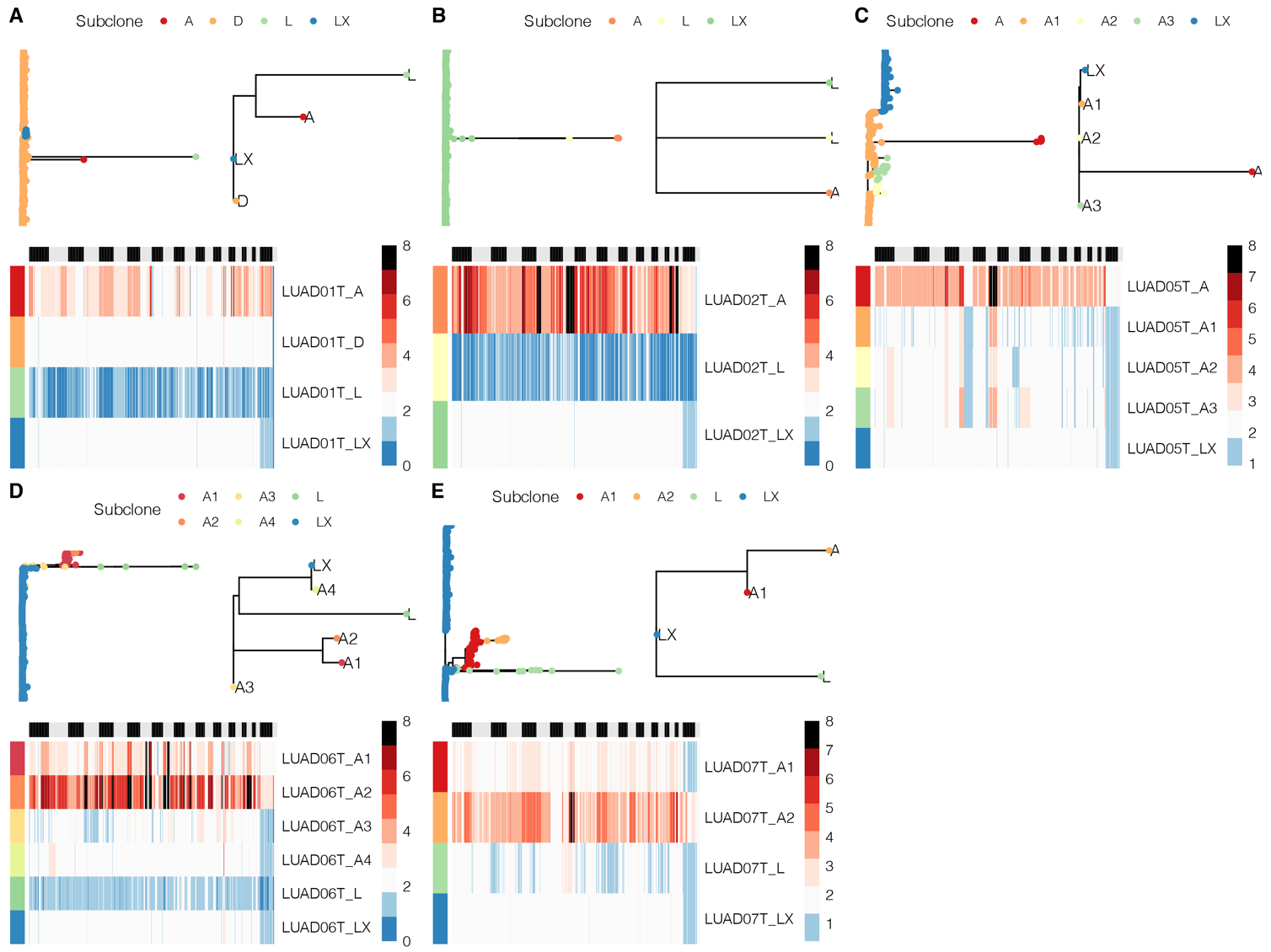


## Figure S7: Copy number evolutionary interpretation of additional five LUAD lung tumors.

(A-E) The copy number evolutionary analysis results for LUAD01T-LUAD02T and LUAD05T-LUAD07T. The top layer comprises the phylogeny tree derived from cell-level CNV profiles and subclone-level consensus CNV profiles. The bottom layer shows the heatmap of the subclone-level consensus CNV profile across the genome. (A) shows subclones D and LX may derive from a PCNE process in LUAD01T. Minor subclones A (one cell) and lost L (one cell) were also discovered. (B) demonstrates LUAD02T comprises a major subclone LX and two minors (A with three cells and L with one cell). (C) In LUAD05T, the PCNE model may generate three subclones LX, A, and A1, then subclone A2 and A3 may be derived from A1 in BCNE. (D) illustrates LUAD06T acquiring three subclones LX, A1, and A3 on a possible PCNE event, followed by a possible BCNE process where A1 evolves to A2. Minor subclones A3 (four cells) and L (four cells) were detected as well. (E) depicts in LUAD07T, subclones A2 and LX may be derived from a PCNE process, then LX may evolve to A1 and L under a BCNE process. CNV: copy number variation. LUAD: lung adenocarcinoma. LUSC: squamous cell cancer. SCLC: small-cell lung cancer. PCNE: punctuated copy number evolution. BCNE: branching copy number evolution. N: normal. A: amplification. D: diploid. L: loss. LX: loss on chromosome X.


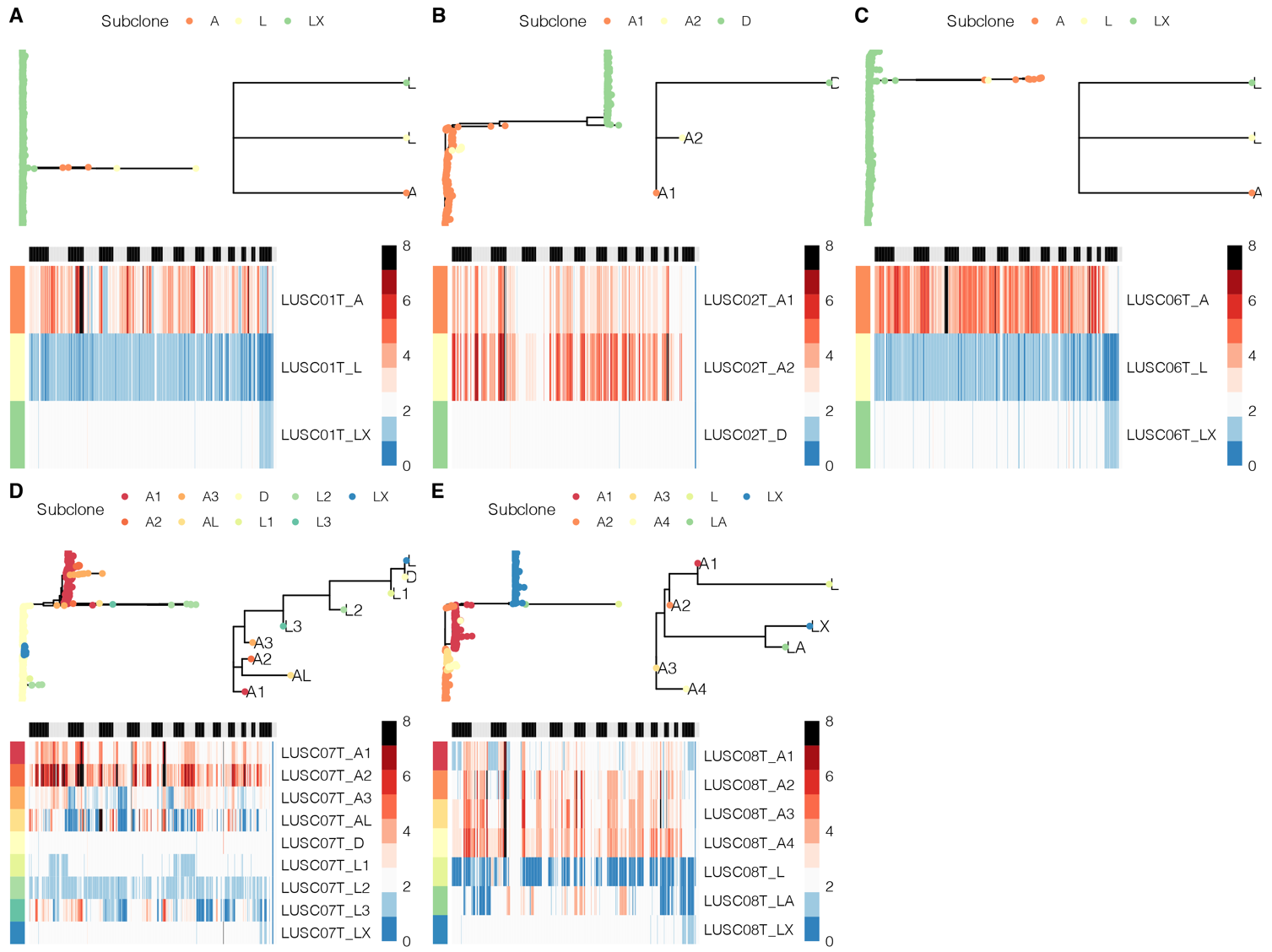


## Figure S8: Copy number evolutionary interpretation of additional five LUSC lung tumors.

(A-E) The copy number evolutionary analysis results for LUSC01T-LUSC02T and LUSC06T-LUSC08T. The

top layer comprises the phylogeny tree derived from cell-level CNV profiles and subclone-level consensus CNV profiles. The bottom layer shows the heatmap of the subclone-level consensus CNV profile across the genome. (A) LUSC01T consists of a dominant subclone LX. Minor cell subpopulations A (three cells) and L (two cells) were discovered as well. (B) LUSC02T may undergo a PCNE event yielding subclones D and A1, then A1 may derive to A2 with seven cells in a branch. (C) Subclones A and LX may be derived from a PCNE model in LUSC06T, minor cell group L with two cells also discovered. (D) In LUSC07T, PCNE may generate subclones LX, D, and A1, followed by a possible BCNE process where A1 evolves to A3. Some minor subclones also were detected (L1 with one cell, L2 with nine cells, L3 with one cell, A2 with nine cells, and AL with one cell). (E) illustrates LUSC08T generates three subclones LX, A1, and A2 under a possible PCNE event, then subclones A3, and A4 may be derived from A2 in a BCNE. CNV: copy number variation. LUAD: lung adenocarcinoma. LUSC: squamous cell cancer. SCLC: small-cell lung cancer. PCNE: punctuated copy number evolution. BCNE: branching copy number evolution. N: normal. A: amplification. D: diploid. L: loss. LX: loss on chromosome X.


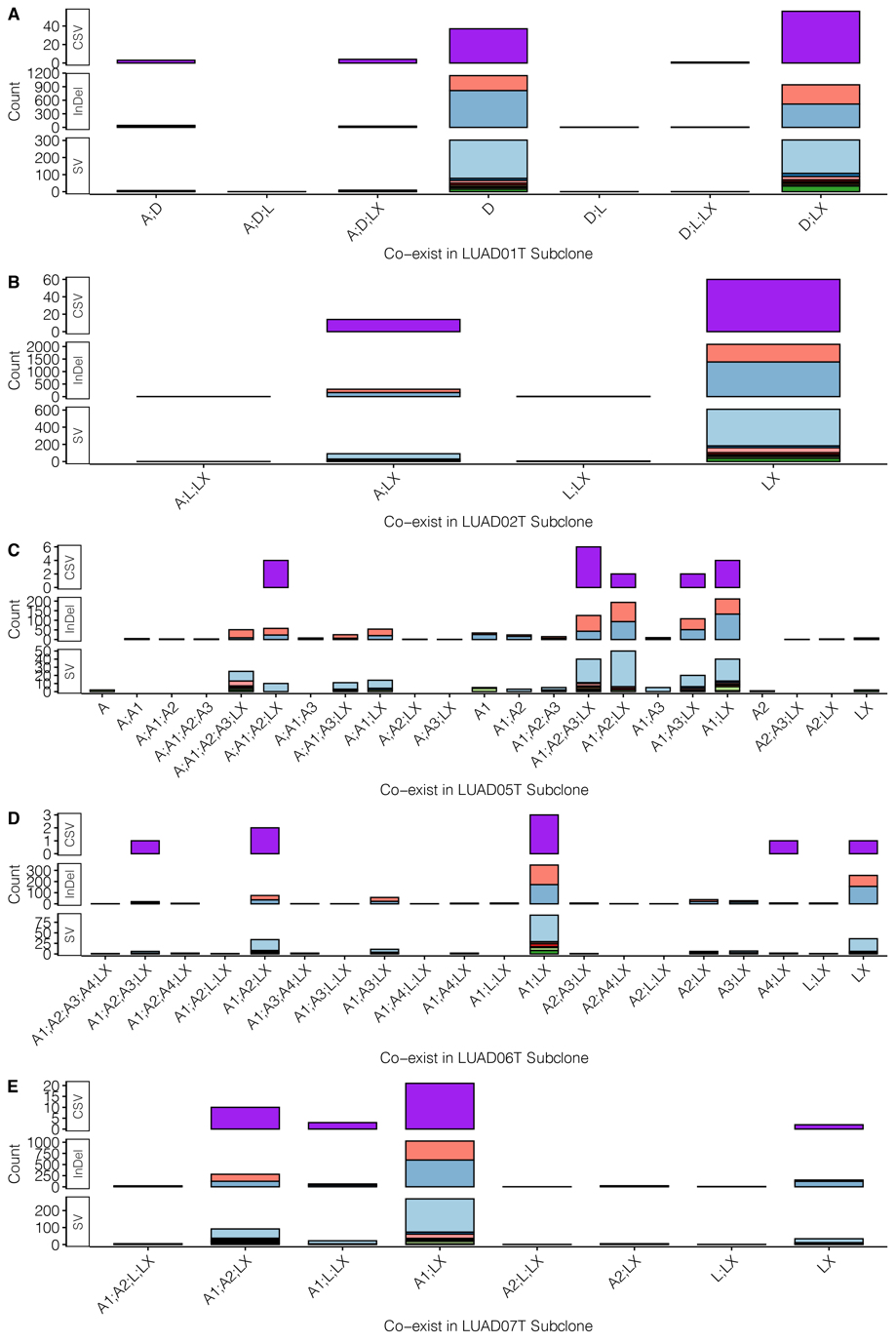


## Figure S9: The frequency of genome aberrations co-existed in multiple subclones for additional five LUAD tumors.

(A-E) The frequency of genome aberrations co-existed in multiple subclones for LUAD01T-LUAD02T and

LUAD05T-LUAD07T. The top, middle, and bottom layer presents InDel, SV, and cSV, respectively. (A) In LUAD01T, subclones D and LX derived from a PCNE process share 44.89% genetic alterations. Subclone D itself has 51.24% distinct alterations. (B) demonstrates 86.73% alterations exclusively occur in the major subclone LX of LUAD02T, and minor subclone A (three cells) shares 12.58% alterations with LX. (C) In LUAD05T, the PCNE model generated subclones LX, A1, A2, and A3 has lots of common alterations (21.37% for A1 and LX; 10.90% for A1, A3, and LX; 20.54% for A1, A2, and LX; 14.33% for A1, A2, A3, and LX). (D) In LUAD06T, subclones LX and A1 acquired from a PCNE event share 40.98% common alterations, LX itself has 27.01% exclusive genetic alterations. (E) depicted in LUAD07T, subclones A1 and LX derived from a PCNE process have 64.32% common genetic alterations, then A2 derived from a BCNE process share 18.72% alterations with subclones A1 and LX as well. CNV: copy number variation. LUAD: lung adenocarcinoma. PCNE: punctuated copy number evolution. BCNE: branching copy number evolution. N: normal. A: amplification. D: diploid. L: loss. LX: loss on chromosome X.


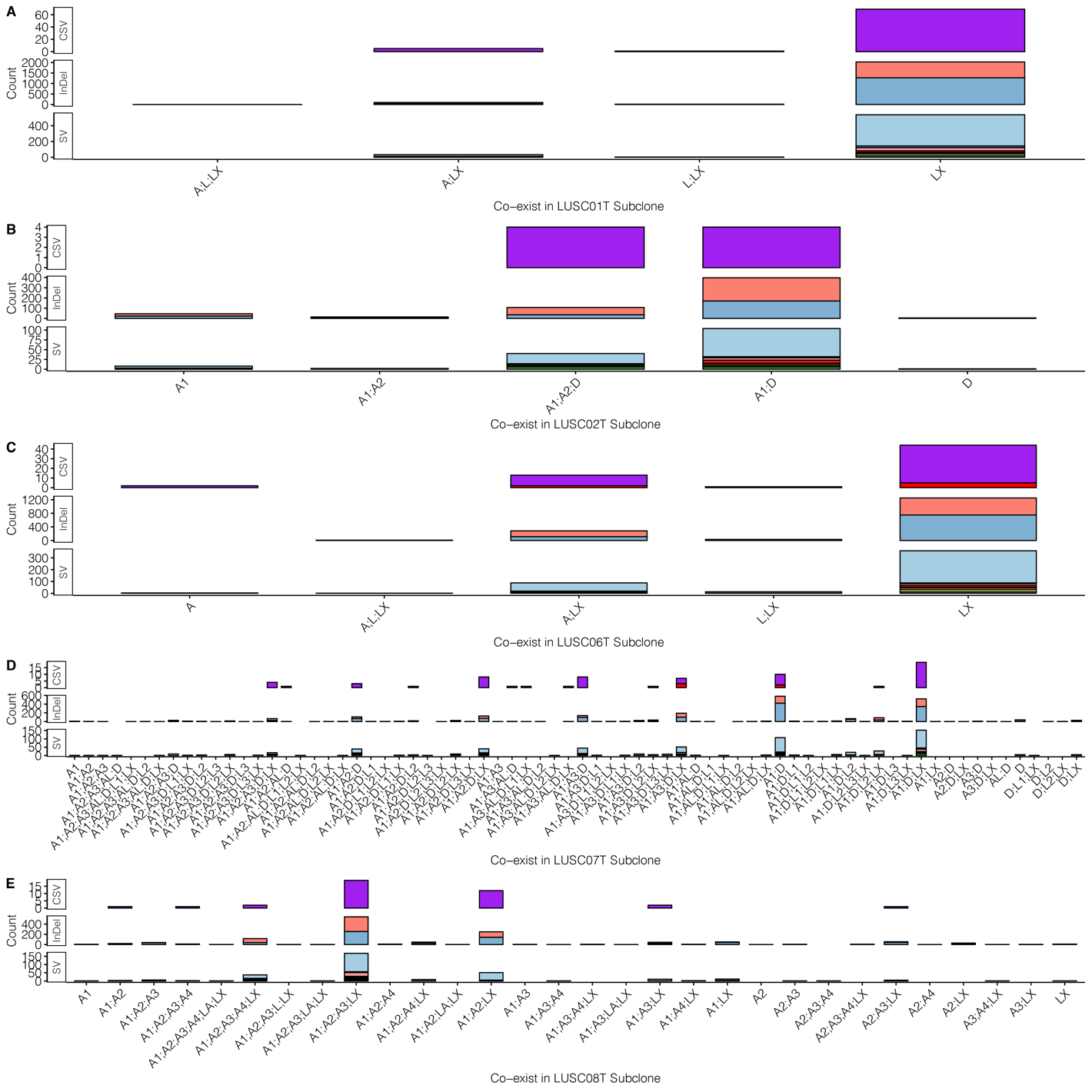


## Figure S10: The frequency of genome aberrations co-existed in multiple subclones for additional five LUSC tumors.

(A-E) The frequency of genome aberrations co-existed in multiple subclones for LUSC01T-LUSC02T and

LUSC06T-LUSC08T. The top, middle, and bottom layer presents InDel, SV, and cSV, respectively. (A) In LUSC01T, the dominant subclone LX has 94.45% distinct genetic alterations. (B) In LUSC02T, subclones D and A1 yielded by PCNE have 68.89% common alterations. Minor subclone A1 with seven cells also share 20.60% genetic alterations with subclone A1 and D. (C) In LUSC06T, subclones A and LX derived from a PCNE model share 18.26% common alterations. Subclone LX itself has 78.70% exclusive alterations. (D) In LUSC07T, PCNE generated subclones LX, D, and A1 have 23.53% common alterations. Subclones A1 and D have 23.74% common alterations as well. (E) In LUSC08T, subclone LX, A1, A2, A3 derived from PCNE share a large proportion of genetic alterations (44.97% for A1, A2, A3, and LX; 19.27% for A1, A2, and LX). CNV: copy number variation. LUSC: squamous cell cancer. PCNE: punctuated copy number evolution. BCNE: branching copy number evolution. N: normal. A: amplification. D: diploid. L: loss. LX: loss on chromosome X.


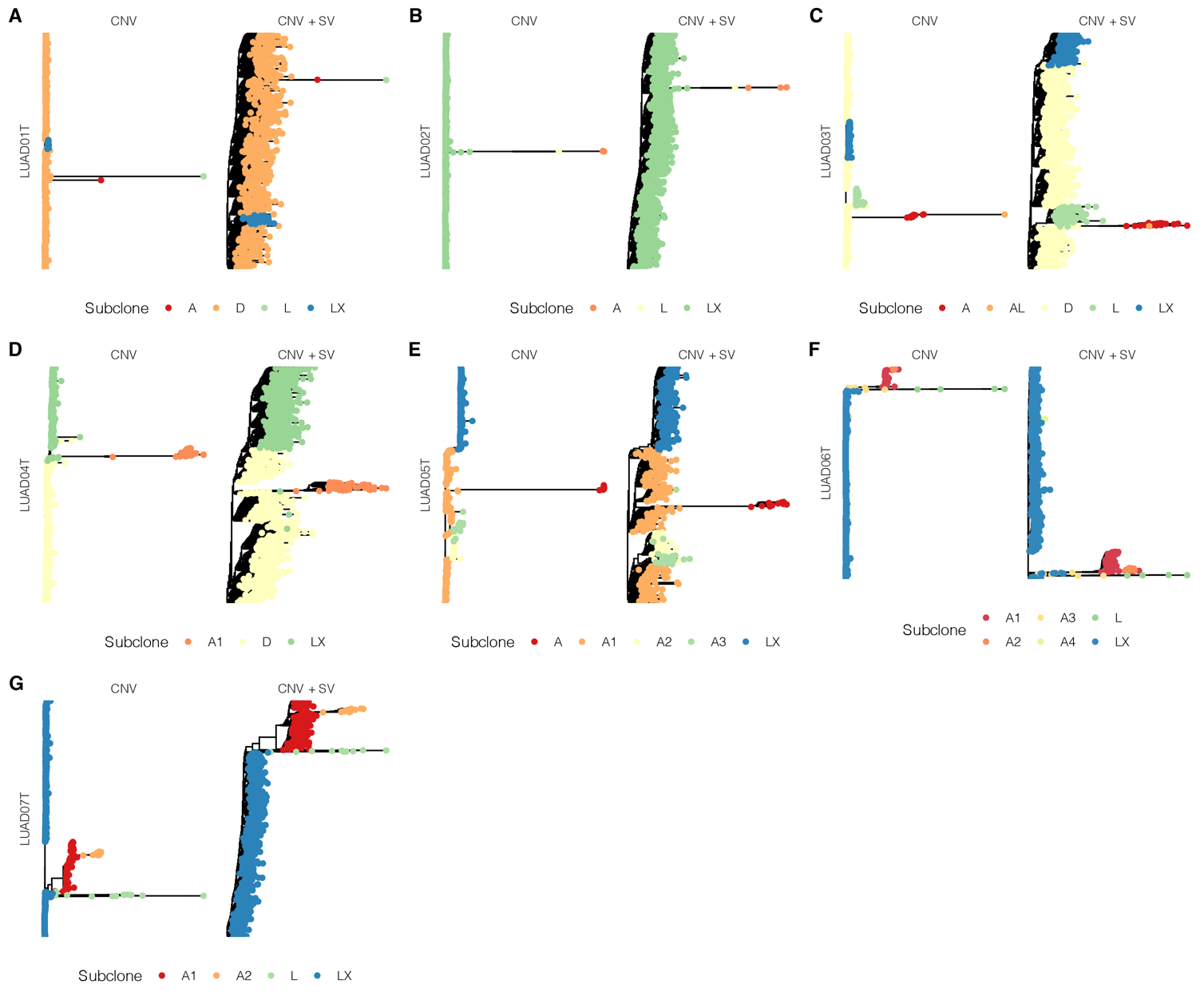


## Figure S11: Copy number and structural variation evolutionary analysis of seven LUAD lung tumors.

(A-G) The copy number and structural variation evolutionary analysis results for LUAD01T-LUAD07T. The left phylogeny tree derived from cell-level CNV profiles and the right phylogenetic tree constructed by CNV and SV events. The dot represents a single cell and is colored with a subclone. CNV: copy number variation. SV: structure variation. LUAD: lung adenocarcinoma. N: normal. A: amplification. D: diploid. L: loss. LX: loss on chromosome X.


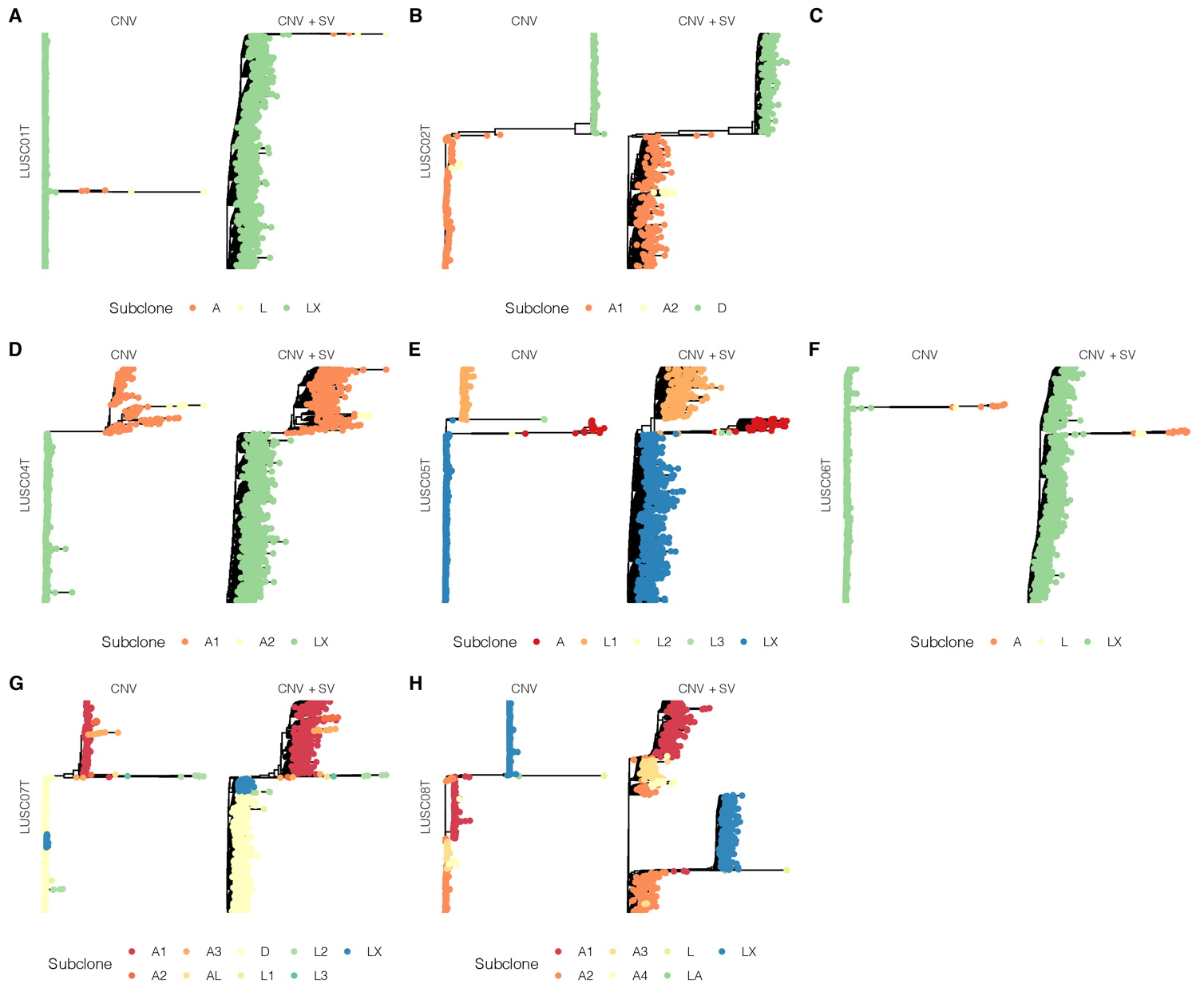


## Figure S12: Copy number and structural variation evolutionary analysis of seven LUSC lung tumors.

(A-G) The copy number and structural variation evolutionary analysis results for LUSC01T-LUSC02T and LUSC04T-LUSC08T. The left phylogeny tree derived from cell-level CNV profiles and the right phylogenetic tree constructed by CNV and SV events. The dot represents a single cell and is colored with a subclone. CNV: copy number variation. SV: structure variation. LUSC: squamous cell cancer. N: normal. A: amplification. D: diploid. L: loss. LX: loss on chromosome X.


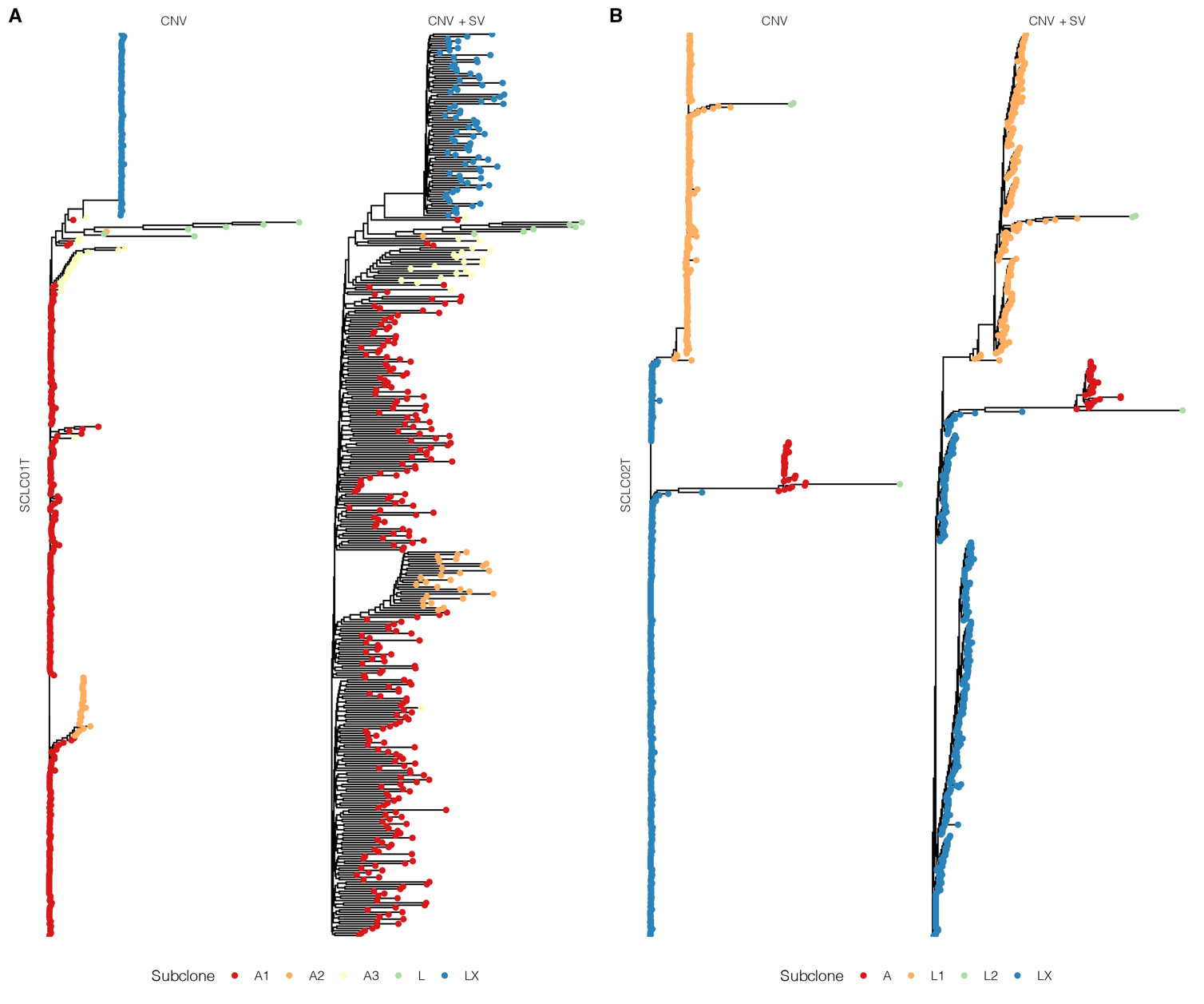


## Figure S13: Copy number and structural variation evolutionary analysis of two SCLC lung tumors.

(A-B) The copy number and structural variation evolutionary analysis results for SCLC01T and SCLC02T. The left phylogeny tree derived from cell-level CNV profiles and the right phylogenetic tree constructed by CNV and SV events. The dot represents a single cell and is colored with a subclone. CNV: copy number variation. SV: structure variation. SCLC: small-cell lung cancer. N: normal. A: amplification. D: diploid. L: loss. LX: loss on chromosome X.


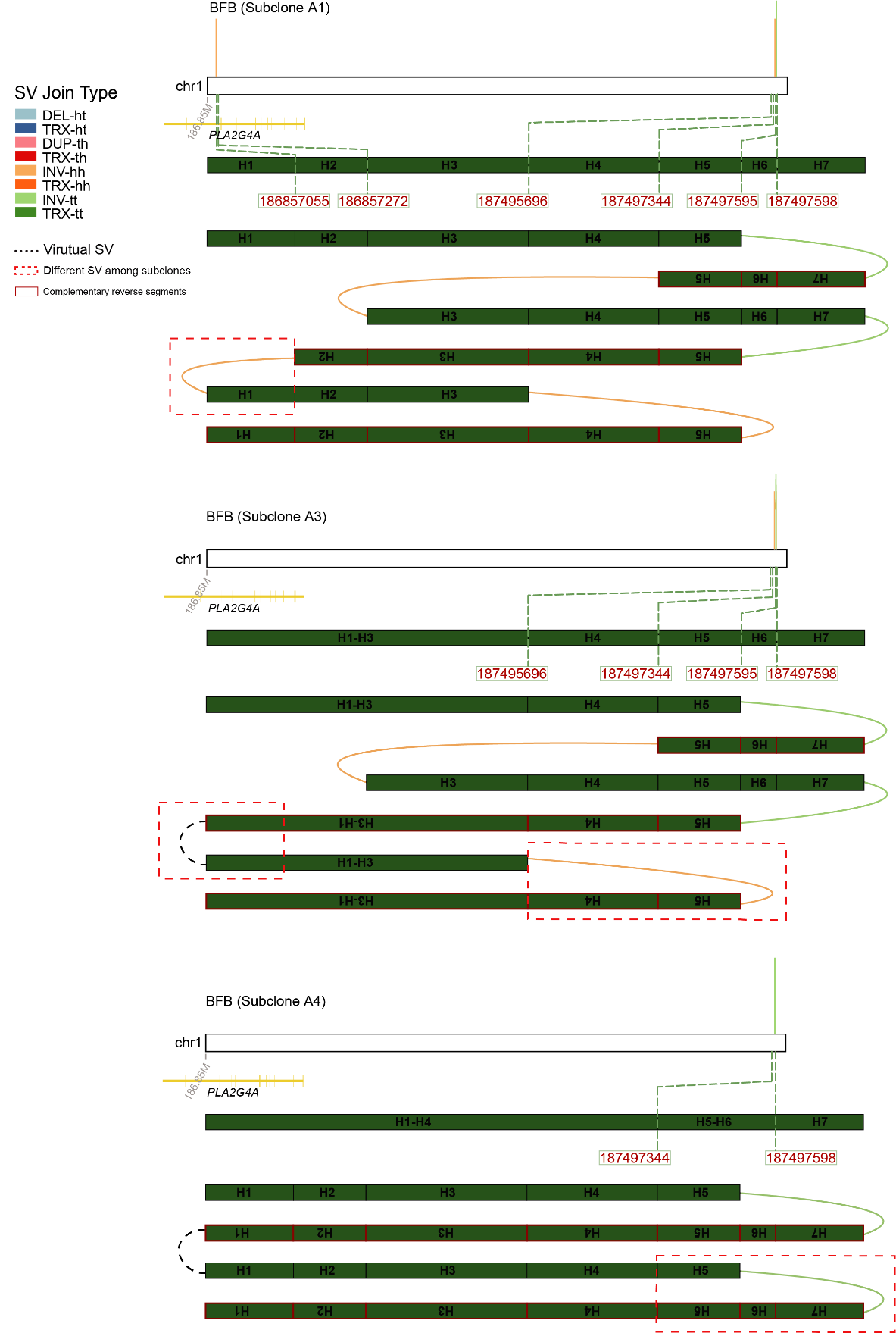


## Figure S14: Complex BFB event on chr1 for LUSC08T.

The breakpoints of four FBIs split the local genome region of chr1 in subclone A1 into seven segments. Based

on the CN profile of subclone A1, Ambigram connects all four SVs in the rearrangement structure derived from four BFB cycles. With one FBI missing in subclone A3, the reconstructed BFB structure, similar to that of subclone A1, is derived from four BFB cycles. Ambigram infers a virtual FBI on segment H1 of subclone A3 based on integrated FBI and CN information of both subclones. Subclone A4 only contains one FBI, the reconstructed BFB structure is different from subclone A1 and A3, derived from two BFB cycles. The yellow horizontal lines represent gene annotation. FBI: fold-back inversion. CN: copy number.


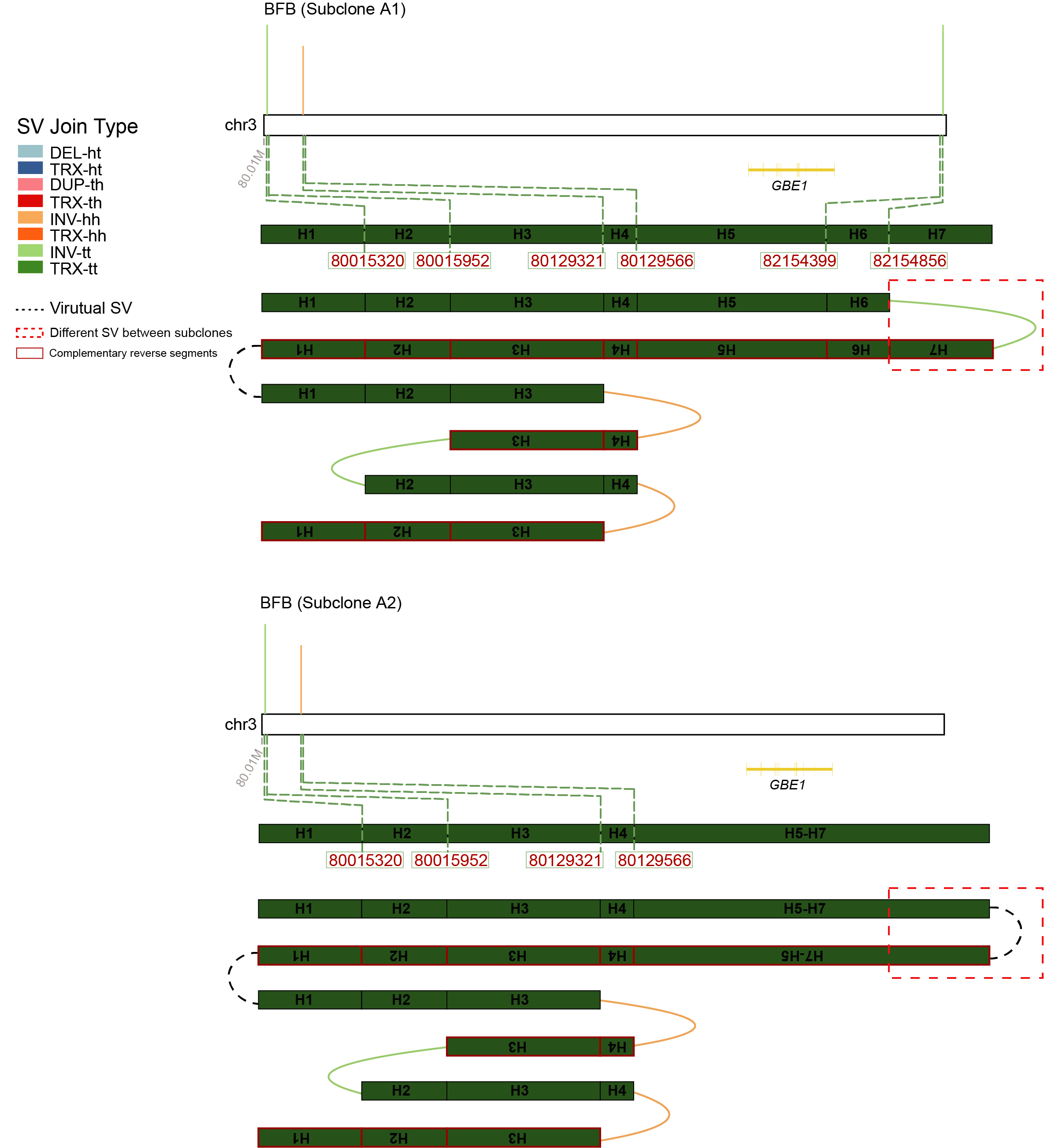


## Figure S15: Complex BFB event on chr3 for SCLC01T.

The breakpoints of three FBIs split the local genome region of chr3 in subclone A1 into seven segments. Based

on the CN profile of subclone A1, Amibgram connects all three SVs in the rearrangement structure derived from four BFB cycles. With one FBI missing in subclone A2, the reconstructed BFB structure, similar to that of subclone A1, is derived from four BFB cycles. Ambigram infers a virtual FBI on segment H7 of subclone A2 based on integrated FBI and CN information of both subclones. The yellow horizontal lines represent gene annotation. FBI: fold-back inversion. CN: copy number.


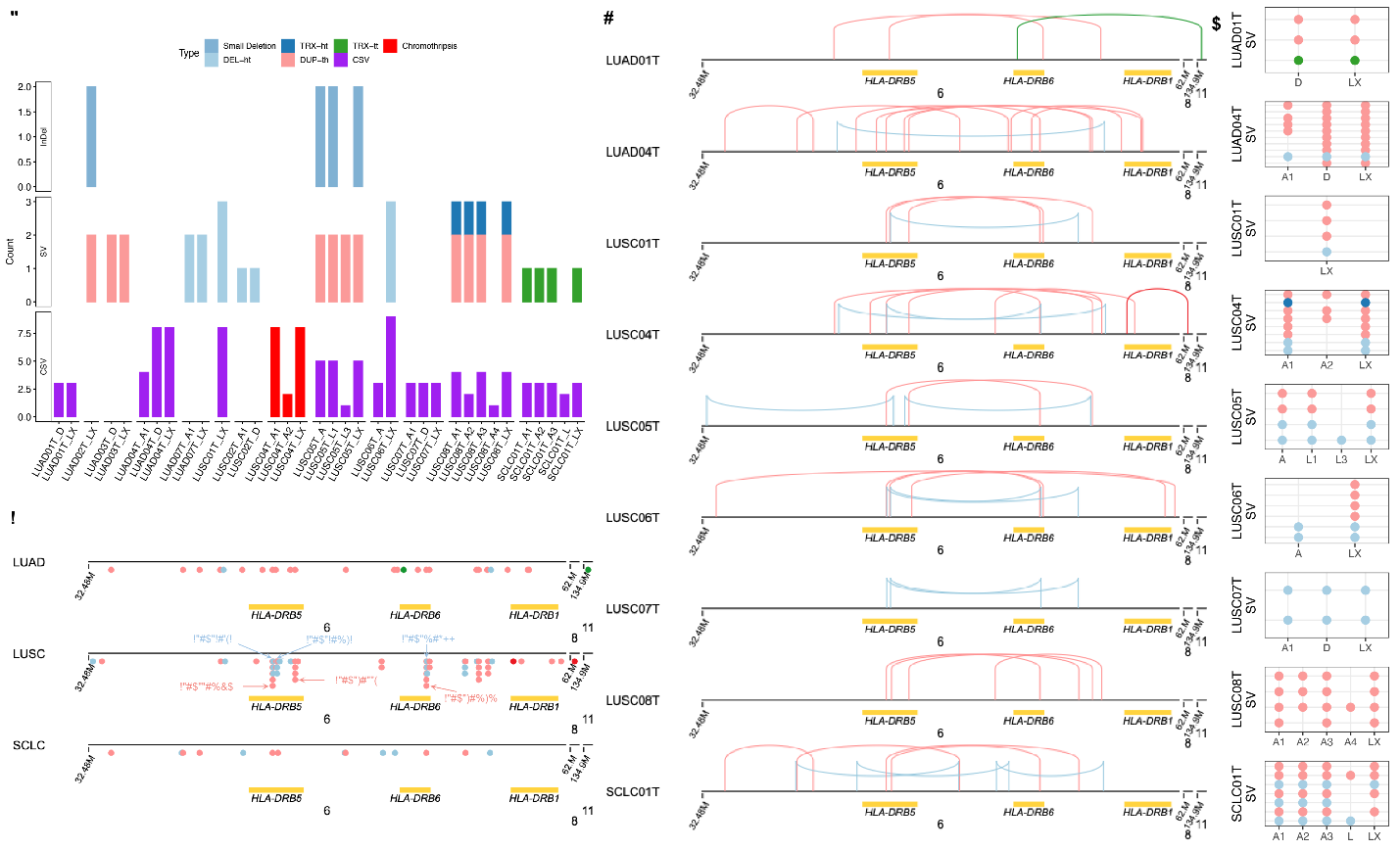


## Figure S16. Subclone-level heterogeneity of complex rearrangements on MHC II genes.

(A) The number of InDel (small insertion and deletion), SV (DEL-ht, TRX-ht, DUP-th, TRX-th, INV-hh, TRX-hh, INV-tt, and TRX-tt), and cSV (Chromothripsis and other cSV) hit HLA genes in each tumor subclone. (B) The occurrence of SV breakpoints on genes HLA-DRB5, HLA-DRB6, and HLA-DRB1 for LUAD, LUSC, and SCLC tumors. A dot represents that an SV breakpoint occurs in one sample, colored by its SV join type. (C) Illustration of a complex rearrangement related to the HLA-DRB genes for LUAD01T, LUAD04T, LUSC01T, LUSC04T-LUSC08T, and SCLC01T. (D) The subclone-level occurrence of the corresponding SV in (C) for LUAD01T, LUAD04T, LUSC01T, LUSC04T-LUSC08T, and SCLC01T. The dot signifies the occurrence of the SV event in the tumor subclone, and colored with its join type.


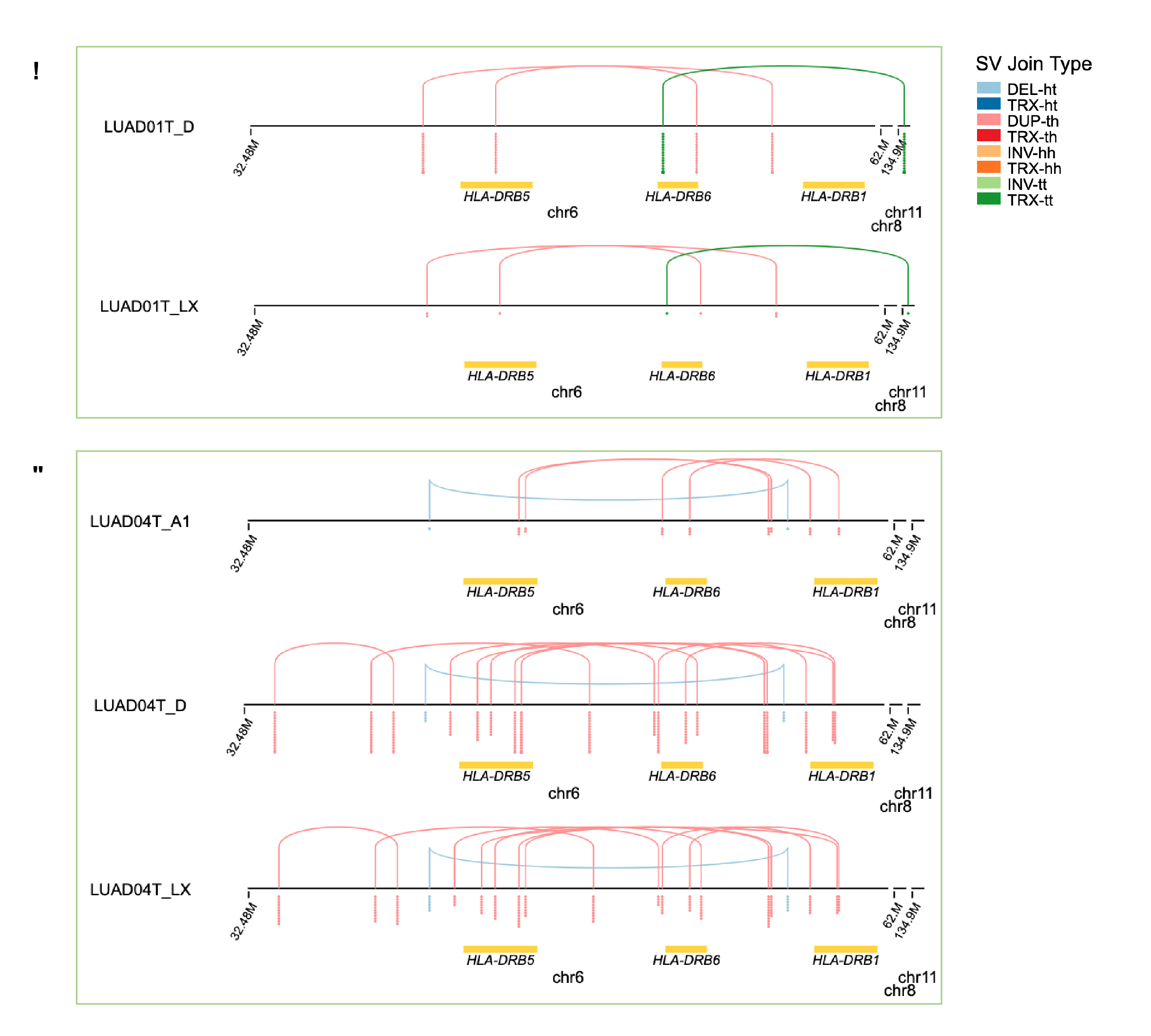


## Figure S17: The subclone-level of HLA-DRB genes associated complex rearrangements in LUAD tumors.

(A-B) The subclone-level of HLA-DRB genes associated with complex rearrangements in LUAD01T and LUAD04T. The top layer illustrates the SV breakpoints and links. The middle layer shows the SV break- points, each dot refers to one supported split read colored with the SV type. SV: structure variation. LUAD: lung adenocarcinoma. N: normal. A: amplification. D: diploid. L: loss. LX: loss on chromosome X.


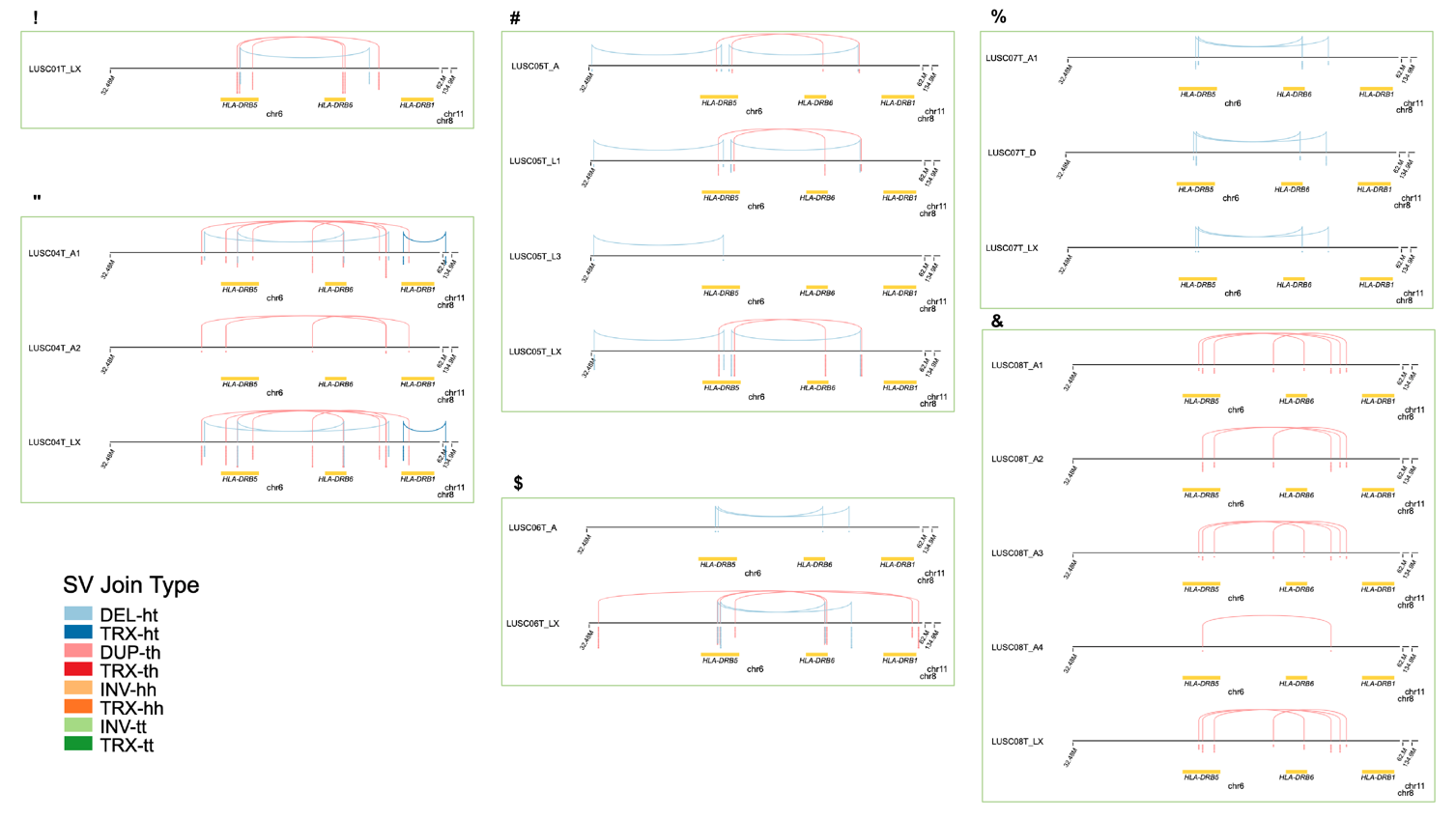


## Figure S18: The subclone-level of HLA-DRB genes associated with complex rearrangements in LUSC tumors.

(A-F) The subclone-level of HLA-DRB genes associated with complex rearrangements in LUSC01T and LUSC04T-LUSC08T. The top layer illustrates the SV breakpoints and links. The middle layer shows the SV breakpoints, each dot refers to one supported split read colored with the SV type. SV: structure variation. LUSC: squamous cell cancer. N: normal. A: amplification. D: diploid. L: loss. LX: loss on chromosome X.


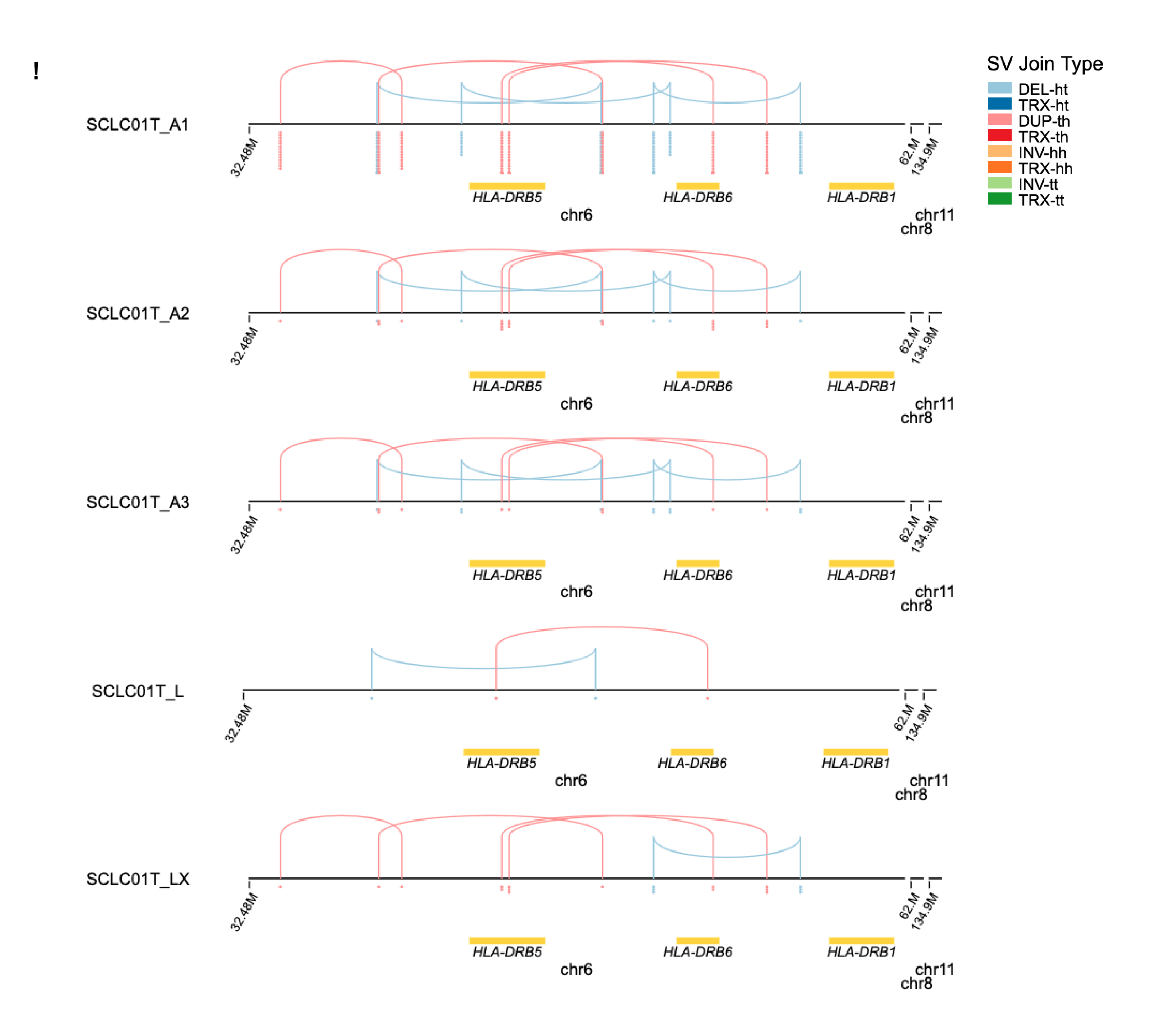


## Figure S19: The subclone-level of HLA-DRB genes associated with complex rearrangements in SCLC tumors.

(A) The subclone-level of HLA-DRB genes associated with complex rearrangements in SCLC01T. The top layer illustrates the SV breakpoints and links. The middle layer shows the SV breakpoints, each dot refers to one supported split read colored with the SV type. SV: structure variation. SCLC: small-cell lung cancer. N: normal. A: amplification. D: diploid. L: loss. LX: loss on chromosome X.


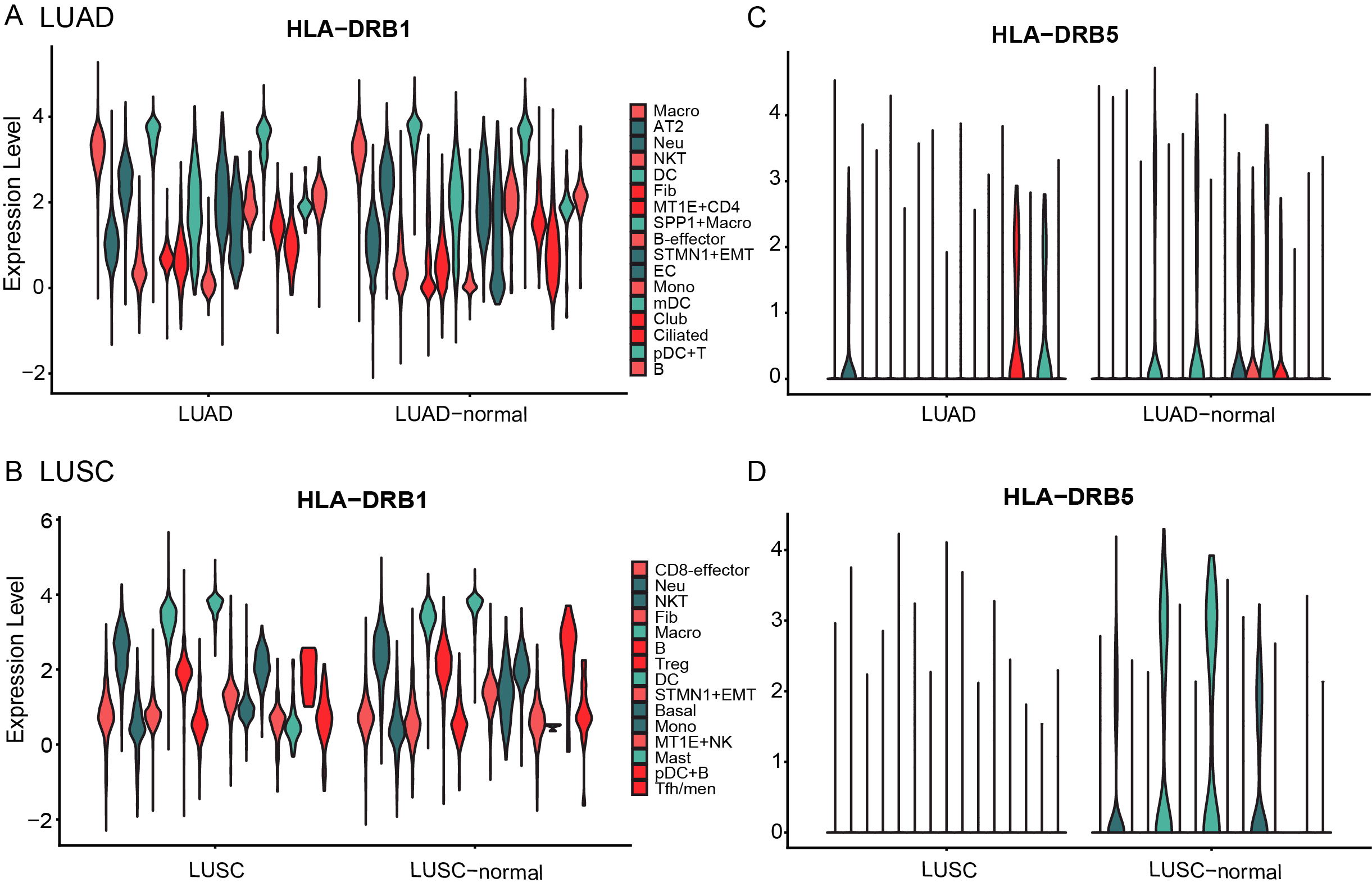


## Figure S20: The expression level of HLA-DRB1 and HLA-DRB5 in additional LUAD and LUSC case-control cohorts.

(A-B) The expression level of HLA-DRB1 in additional LUAD and LUSC case-control cohorts. (C-D) The expression level of HLA-DRB5 in additional LUAD and LUSC case-control cohorts. LUAD: lung adenocarcinoma. LUSC: squamous cell cancer. SCLC: small-cell lung cancer. AT2: alveolar type II cells. Ciliated: ciliated cells. Club: club cells. EC: endothelial cells. Fib: fibroblasts. B-effector: plasma B cells. B: B cells. MT1E+CD4: MT1E high CD4+ T cells. NKT: natural killer T cells. Macro: macrophage. Neu: neutrophil. Mono: Monocyte. STMN1+EMT: STMN1+epithelial mesenchymal transition cells. SPP1+Macro: SPP1 high macrophage. pDC+T, mDC, and DC: 3 dendritic cells. Basal: basal cells. Mast: mast cells. Treg: regular T cells. CD8+effector: CD8+effector T cells. DC, pDC+B: 2 dendritic cells.

# References

1. Siegel, R. L., Miller, K. D., Fuchs, H. E. & Jemal, A. Cancer Statistics, 2021. *CA Cancer J Clin* **71**, (2021).

2. Travis, W. D. Pathology of Lung Cancer. *Clinics in Chest Medicine* vol. 32 669–692 Preprint at https://doi.org/10.1016/j.ccm.2011.08.005 (2011).

3. Grass, G. D., Naghavi, A. O., Abuodeh, Y. A., Perez, B. A. & Dilling, T. J. Analysis of Relapse Events After Definitive Chemoradiotherapy in Locally Advanced Non–Small-Cell Lung Cancer Patients. *Clin Lung Cancer* **20**, (2019).

4. Yang, H., Jin, T., Li, M., Xue, J. & Lu, B. Synergistic effect of immunotherapy and radiotherapy in non-small cell lung cancer: current clinical trials and prospective challenges. *Precis Clin Med* **2**, 57–70 (2019).

5. van Meerbeeck, J. P., Fennell, D. A. & de Ruysscher, D. K. M. Small-cell lung cancer. in *The Lancet* vol. 378 1741–1755 (Elsevier B.V., 2011).

6. Jamal-Hanjani, M. *et al.* Tracking the Evolution of Non–Small-Cell Lung Cancer. *New England Journal of Medicine* **376**, 2109–2121 (2017).

7. Wu, C. *et al.* High discrepancy of driver mutations in patients with NSCLC and synchronous multiple lung ground-glass nodules. *Journal of Thoracic Oncology* **10**, (2015).

8. Velazquez-Villarreal, E. I. *et al.* Single-cell sequencing of genomic DNA resolves sub-clonal heterogeneity in a melanoma cell line. *Commun Biol* **3**, 1–8 (2020).

9. Navin, N. *et al.* Tumour evolution inferred by single-cell sequencing. *Nature* **472**, 90–95 (2011).

10. Lan, F., Demaree, B., Ahmed, N. & Abate, A. R. Single-cell genome sequencing at ultra-high-throughput with microfluidic droplet barcoding. *Nat Biotechnol* **35**, (2017).

11. Andor, N. *et al.* Joint single cell DNA-seq and RNA-seq of gastric cancer cell lines reveals rules of in vitro evolution. *NAR Genom Bioinform* **2**, 1–13 (2020).

12. Martelotto, L. G. *et al.* Whole-genome single-cell copy number profiling from formalin-fixed paraffin-embedded samples. *Nat Med* **23**, 376–385 (2017).

13. Casasent, A. K. *et al.* Multiclonal Invasion in Breast Tumors Identified by Topographic Single Cell Sequencing. *Cell* **172**, 205-217.e12 (2018).

14. Kim, C. *et al.* Chemoresistance Evolution in Triple-Negative Breast Cancer Delineated by Single-Cell Sequencing. *Cell* **173**, 879-893.e13 (2018).

15. Gawad, C., Koh, W. & Quake, S. R. Dissecting the clonal origins of childhood acute lymphoblastic leukemia by single-cell genomics. *Proc Natl Acad Sci U S A* **111**, (2014).

16. Eastburn, D. J. *et al.* Abstract 5348: Single-cell analysis of mutational heterogeneity in acute myeloid leukemia tumors with high-throughput droplet microfluidics. 5348–5348 (2018) doi:10.1158/1538-7445.am2018-5348.

17. Leung, M. L. *et al.* Single-cell DNA sequencing reveals a latedissemination model in metastatic colorectal cancer. *Genome Res* **27**, (2017).

18. Gao, R. *et al.* Punctuated copy number evolution and clonal stasis in triple-negative breast cancer. *Nat Genet* **48**, 1119–1130 (2016).

19. Wang, Y. *et al.* Clonal evolution in breast cancer revealed by single nucleus genome sequencing. *Nature* **512**, 155–160 (2014).

20. Minussi, D. C. *et al.* Breast tumours maintain a reservoir of subclonal diversity during expansion. *Nature* **592**, 302–308 (2021).

21. Ni, X. *et al.* Reproducible copy number variation patterns among single circulating tumor cells of lung cancer patients. *Proc Natl Acad Sci U S A* **110**, 21083–21088 (2013).

22. Carter, L. *et al.* Molecular analysis of circulating tumor cells identifies distinct copy-number profiles in patients with chemosensitive and chemorefractory small-cell lung cancer. *Nat Med* **23**, 114–119 (2017).

23. Su, Z. *et al.* Inferring the evolution and progression of small-cell lung cancer by single-cell sequencing of circulating tumor cells. *Clinical Cancer Research* **25**, (2019).

24. Lee, J. J. K. *et al.* Tracing Oncogene Rearrangements in the Mutational History of Lung Adenocarcinoma. *Cell* **177**, 1842-1857.e21 (2019).

25. Luebeck, J. *et al.* AmpliconReconstructor integrates NGS and optical mapping to resolve the complex structures of focal amplifications. *Nat Commun* **11**, 1–14 (2020).

26. Kitada, K. & Yamasaki, T. The complicated copy number alterations in chromosome 7 of a lung cancer cell line is explained by a model based on repeated breakage-fusion-bridge cycles. *Cancer Genet Cytogenet* **185**, 11–19 (2008).

27. Chalishazar, M. D. *et al.* MYC-driven small-cell lung cancer is metabolically distinct and vulnerable to arginine depletion. *Clinical Cancer Research* **25**, 5107–5121 (2019).

28. Yi, K. & Ju, Y. S. Patterns and mechanisms of structural variations in human cancer. *Exp Mol Med* **50**, 98 (2018).

29. Cortés-Ciriano, I. *et al.* Comprehensive analysis of chromothripsis in 2,658 human cancers using whole-genome sequencing. *Nat Genet* **52**, 331–341 (2020).

30. Stephens, P. J. *et al.* Massive genomic rearrangement acquired in a single catastrophic event during cancer development. *Cell* **144**, 27–40 (2011).

31. George, J. *et al.* Comprehensive genomic profiles of small cell lung cancer. *Nature* **524**, 47–53 (2015).

32. Marty, R., Thompson, W. K., Salem, R. M., Zanetti, M. & Carter, H. Evolutionary Pressure against MHC Class II Binding Cancer Mutations. *Cell* **175**, 416-428.e13 (2018).

33. Zhang, Y. *et al.* Global analysis of chromosome 1 genes among patients with lung adenocarcinoma, squamous carcinoma, large-cell carcinoma, small-cell carcinoma, or non-cancer. *Cancer and Metastasis Reviews* **34**, (2015).

34. Chou, R. H. *et al.* Suppression of the invasion and migration of cancer cells by SERPINB family genes and their derived peptides. *Oncol Rep* **27**, (2012).

35. Ranzani, M. *et al.* Revisiting olfactory receptors as putative drivers of cancer. *Wellcome Open Res* **2**, (2017).

36. Zeng, L. *et al.* CHSY1 promoted proliferation and suppressed apoptosis in colorectal cancer through regulation of the nfκb and/or caspase-3/7 signaling pathway. *Oncol Lett* **16**, 6140–6146 (2018).

37. Zhang, N. *et al.* The study on copy number alteration of clear cell renal cancer in Chinese population. *J Cancer* **11**, 16–24 (2020).

38. Zhang, X., Cheng, D., Liu, Y., Wu, Y. & He, Z. <p>Gephyrin suppresses lung squamous cell carcinoma development by reducing mTOR pathway activation</p>. *Cancer Manag Res* **Volume 11**, 5333–5341 (2019).

39. MUC3A induces PD-L1 and reduces tyrosine kinase inhibitors effects in EGFR-mutant non-small cell lung cancer [Abstract]. https://www.ijbs.com/v17p1671.

40. Hu, D. D., Chen, H. L., Lou, L. M., Zhang, H. & Yang, G. L. SKA3 promotes lung adenocarcinoma metastasis through the EGFR-PI3K-Akt axis. *Biosci Rep* **40**, 20194335 (2020).

41. Jiao, X. *et al.* Gene rearrangements in hormone receptor negative breast cancers revealed by mate pair sequencing. *BMC Genomics* **14**, (2013).

42. Gai, M., Bo, Q. & Qi, L. Epigenetic down-regulated DDX10 promotes cell proliferation through Akt/NF-κB pathway in ovarian cancer. *Biochem Biophys Res Commun* **469**, 1000–1005 (2016).

43. Yang, Y. *et al.* DEPDC1B enhances migration and invasion of non-small cell lung cancer cells via activating Wnt/β-catenin signaling. *Biochem Biophys Res Commun* **450**, 899–905 (2014).

44. Tian, H. *et al.* Raf1 is a prognostic factor for progression in patients with non-small cell lung cancer after radiotherapy. *Oncol Rep* **39**, 1966–1974 (2018).

45. Xu, M. *et al.* MED12 exerts an emerging role in actin-mediated cytokinesis via LIMK2/cofilin pathway in NSCLC. *Mol Cancer* **18**, (2019).

46. Zhou, L. *et al.* Association of MUC19 Mutation With Clinical Benefits of Anti-PD-1 Inhibitors in Non-small Cell Lung Cancer. *Front Oncol* **11**, (2021).

47. Hisamatsu, T. *et al.* PRKRA/PACT expression promotes chemoresistance of mucinous ovarian cancer. *Mol Cancer Ther* **18**, 162–172 (2019).

48. Earl, J. *et al.* Evaluation of the 4q32-34 locus in European familial pancreatic cancer. *Cancer Epidemiology Biomarkers and Prevention* **15**, 1948–1955 (2006).

49. Goldstraw, P. *et al.* The IASLC lung cancer staging project: Proposals for revision of the TNM stage groupings in the forthcoming (eighth) edition of the TNM Classification for lung cancer. *Journal of Thoracic Oncology* **11**, 39–51 (2016).

50. Mermel, C. H. *et al.* GISTIC2.0 facilitates sensitive and confident localization of the targets of focal somatic copy-number alteration in human cancers. *Genome Biol* **12**, 1–14 (2011).

51. Wala, J. A. *et al.* SvABA: Genome-wide detection of structural variants and indels by local assembly. *Genome Res* **28**, 581–591 (2018).

52. Geoffroy, V. *et al.* AnnotSV: An integrated tool for structural variations annotation. *Bioinformatics* **34**, (2018).

53. Korbel, J. O. & Campbell, P. J. Criteria for inference of chromothripsis in cancer genomes. *Cell* **152**, 1226–1236 (2013).

54. Price, M. N., Dehal, P. S. & Arkin, A. P. FastTree 2 - Approximately maximum-likelihood trees for large alignments. *PLoS One* **5**, e9490 (2010).

55. Yu, G., Smith, D. K., Zhu, H., Guan, Y. & Lam, T. T. Y. ggtree: an r package for visualization and annotation of phylogenetic trees with their covariates and other associated data. *Methods Ecol Evol* **8**, 28–36 (2017).

# 
